# Research Advances in *Gastrodia elata* Endophytes: Diversity, Secondary Metabolites and Pharmacological Activities

**DOI:** 10.3390/jof12080614

**Published:** 2026-08-15

**Authors:** Tianzhen Xie, Kaize Shen, Wenjian Xia, Wenyun Tan, Congjia Xie, Zhengbiao Zhang, Zhilong Shi, Xin Wei

**Affiliations:** 1 College of Chemistry and Chemical Engineering, Zhaotong University, Zhaotong 657000, China; ztushenkz@163.com (K.S.); 15887962420@163.com (W.X.); 20220053@ztu.edu.cn (W.T.); 20220054@ztu.edu.cn (C.X.); zhengbiaozhang987@163.com (Z.Z.); zhilong32@163.com (Z.S.); 2 Yunnan Key Laboratory of Gastrodia and Fungi Symbiotic Biology of Yunnan Province, Zhaotong University, Zhaotong 657000, China; 3 School of Pharmacy, Guizhou University of Traditional Chinese Medicine, Guiyang 550025, China

**Keywords:** *Gastrodia elata*, endophytes, diversity, secondary metabolites, bioactivity

## Abstract

*Gastrodia elata*, a medicinal and edible plant of the Orchidaceae family, has a long history of medicinal application and extensive development value in China. Endophytes permanently colonize *G. elata* and form a stable long-term symbiotic relationship with the host. The secondary metabolites produced by these fungi possess diverse structures and multiple biological activities, which are important sources for discovering novel bioactive ingredients that provide natural materials for screening and developing medicinal compounds. This article reviewed the research and development progress of endophytes associated with *G. elata*, including endophytes’ sources, biological functions, compound classification and pharmacological activities. The results showed that more than 296 secondary metabolites were reported—mainly terpenoids, polyketides, alkaloids, anthraquinones, phenolics—with prominently characterized antifeedant and antibacterial activities. Our work aims to provide scientific references for the in-depth exploration and efficient utilization of *G. elata* endophytic fungal resources.

## 1. Introduction

*Gastrodia elata*, a perennial herbaceous plant of the genus *Gastrodia* in the family Orchidaceae, is also known as Tianma, Chi jian and Ming Tianma [1,2]. *G. elata*, utilized as a traditional functional food, is primarily distributed across China, Japan, Korea, and Russia. With a long history of use and significant medicinal value, it was included in the list of medicinal–edible homologs in China in 2023 [3]. Its tubers are used medicinally, with effects such as calming the liver and stopping wind, relieving convulsions and dispelling dampness, and replenishing qi and relieving spasms. It is used to treat symptoms such as dizziness, numbness and pain in the limbs, infantile convulsions, hypertension, and otogenic vertigo [3,4,5]. Studies have confirmed that *G. elata* contains a complex chemical composition, and its main components include phenols, polysaccharides, sterols, and organic acids [6,7,8,9,10]. Modern pharmacological studies have shown that the extracts of *G. elata* or its active components possess wide range of biological activities, such as anticancer, antiviral, memory improvement, antioxidant and anti-aging, brain protection, sedative and hypnotic, analgesic, anti-epileptic, anti-vertigo, and antihypertensive and lipid-lowering effects [4,11,12,13]. *G. elata* has neither roots nor leaves, cannot perform photosynthesis, and cannot directly absorb nutrients from the soil. Its growth requires fungi to supply nutrients for survival. After seed germination, the protocorm must form a symbiotic relationship with *Armillaria mellea* to develop normally [14].

Endophytes are a group of microorganisms that live within plant tissues or organs during a certain stage or the entire life cycle of the plant without causing apparent pathogenic symptoms in the host plant. They can promote host plant growth and development, enhance stress resistance, and facilitate the accumulation of secondary metabolites, thereby influencing the host’s growth and development [15]. Moreover, endophytic fungi are highly diverse and can produce secondary metabolites with rich structural types and diverse biological activities [16,17,18]. The metabolic products of these fungi are often identical or similar to those of the host plant [19]. For example, the endophytic fungus *Taxomyces andreanae*, isolated from *Taxus brevifolia* Nutt., can produce paclitaxel, a metabolite identical to that of the host. Therefore, the metabolites of such endophytic fungi are considered a new resource to replace woody medicinal plants that are scarce or have long growth cycles, helping to protect rare and endangered woody medicinal plants and providing new directions and possibilities for future drug development and resource conservation [20]. In recent years, many researchers have isolated various endophytes from *G. elata* using tissue isolation methods and have studied their diversity and functions. Endophytes have significant application value and potential in inducing the production of gastrodin in *G. elata* and improving its stress resistance.

Previously, a large number of investigations have been conducted on *G. elata*, but existing published reviews concerning *G. elata*-associated fungi predominantly focus on obligate symbiotic mycorrhizal fungi such as *Armillaria* and *Mycena*, centering on host fungus nutrient exchange and symbiotic molecular regulatory mechanisms [21]. Meanwhile, general reviews on the pharmacology of *G. elata* mainly concentrate on intrinsic plant derived ingredients rather than metabolites produced by non-symbiotic endophytic fungi [22,23,24,25]. Up to now, few targeted systematic reviews have comprehensively summarized the taxonomic diversity, functional characteristics, secondary metabolites and pharmacological activities of broad spectrum non-symbiotic endophytic fungi isolated from *G. elata*. To fill this clear research gap, this paper presents a systematic and methodical compilation of the diversity, biological functions derived from *G. elata* fungi, along with their associated secondary metabolites and pharmacological effectiveness. This work intends to highlight the resource value of *G. elata* endophytic fungi, lay a theoretical foundation for subsequent mining of novel fungal bioactive compounds, and promote the translational research of these metabolites for disease prevention and treatment.

## 2. Biodiversity of Endophytes Isolated from *G. elata*

**Figure 1:**
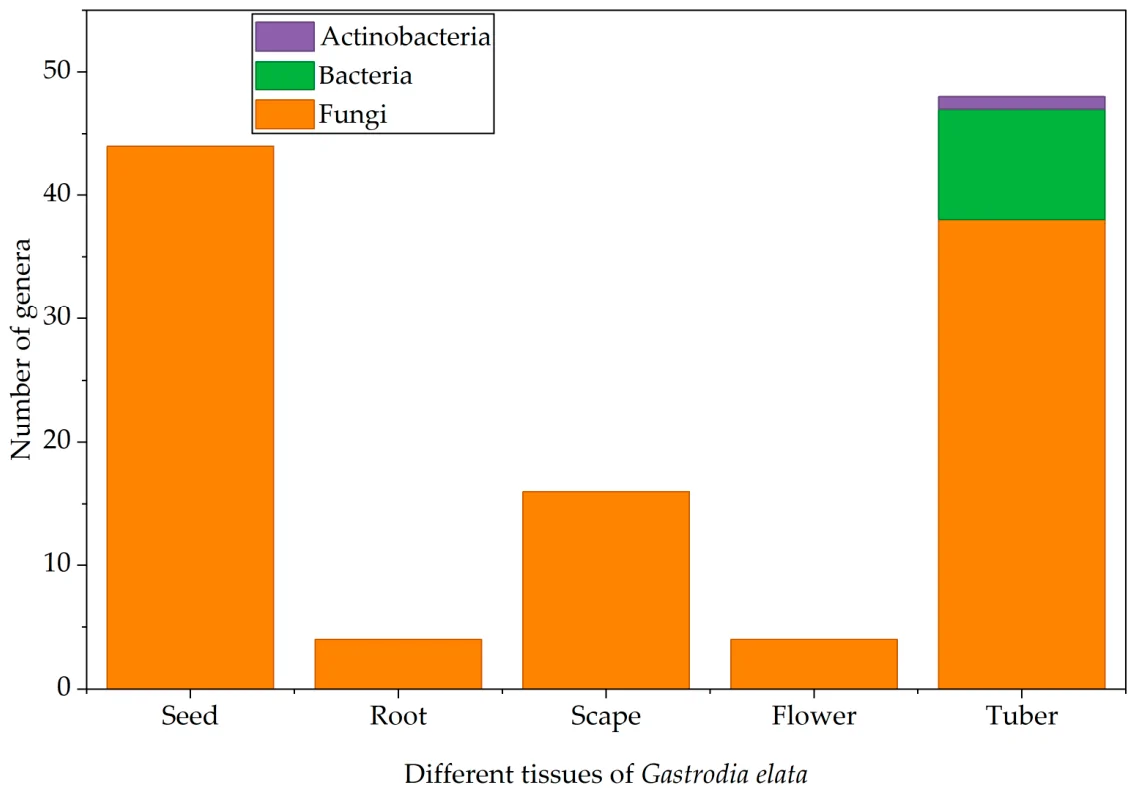
Distribution of fungal, bacterial and actinobacterial genera from various tissues of *G. elata*.

Endophytic microorganisms are widely distributed within different plant tissues and exhibit significant diversity in species [26]. *G. elata* is known for its adaptability and medicinal properties, making it a subject of extensive research, and different ecological environments and tissue parts can influence plant endophytes leading to differences in endophytic microbial communities [27]. According to their types, they can be roughly divided into endophytic bacteria, endophytic fungi, and endophytic actinomycetes [28]. Today, researchers have found that endophytic microorganisms in *G. elata* from different origins and parts are mainly concentrated in the seed and tuber, exhibiting rich species diversity. A total of 85 unique genera of endophytic microorganisms have been reported to be isolated and identified from diverse tissues and organs of *Gastrodia* plants, including 74 genera of endophytic fungi, 9 genera of endophytic bacteria and 2 actinobacterial genera, as shown in Table 1 and Figure 1.

It was reported that a total of 44 endophytic genera were isolated and identified from *G. elata* seeds collected at three distinct locations. Forty-five genera of endophytic fungi have been identified from the seeds of *G. elata*. In 2022, the seeds collected from Zhaotong, and conventional tissue separation and monomer separation methods were used to isolate 22 strains of endophytic fungi, among which two strains of *Schizophyllum commune* and *Irpex lacteus* with rich metabolites were identified by morphological and microscopic observation and ITS sequencing [29,30]. A total of 94 endophytic fungi were isolated from different tissues collected from Guizhou, Shaanxi, Sichuan and Yunnan, from which 62 endophytic fungi were identified and belonged to 4 subphylums, 6 classes, 8 orders, 11 families, and 15 genera. The study found significant differences in the endophytic fungal communities isolated from the same tissue across different regions. The main focus of this study was to describe the differences in species composition and abundance of endophytic fungi in Tianma from different producing areas, with Guizhou’s Dafang Tianma showing the highest abundance and Sichuan’s Guangyuan Tianma having the greatest species diversity [31,32]. A total of 334 strains of endophytic fungi were obtained from 2380 pieces of tissue in the five stages of *Gastrodia* tuber collected from Lianfeng, Yongshan County, Zhaotong, Yunnan. A total of 113, 63, 59, 39 and 60 strains of endophytic fungi were obtained from the five stages from the initial planting stage to the mature stage, respectively. At the genus level, endophytic fungi are mainly composed of *Trichoderma*, *Aspergillus*, *Penicillium* and *Chaetomium* in the initial planting stage; *Penicillium*, *Aspergillus*, *Talaromyces* and *Trichoderma* are dominant in the emergence stage; *Ilyonectria* is dominant in the budding stage; the endophytic fungus *Chaetomium* has become the dominant genus again in the flowering stage; the endophytic fungi *Trichoderma*, *Penicillium*, *Aspergillus* and *Fusarium* are dominant during the mature stage. Diversity analysis showed that the diversity, evenness and richness index of endophytic fungi were the highest during the emergence stage [33].

Five strains of endophytic fungi were isolated from the roots of *G. elata* in Changbai Mountain, Changchun, and three of them were identified as *Trichoderma harzianum*, *Talaromyces stollii*, and *Cryptosporiopsis ericae* through morphological and microscopic observation and ITS sequencing. At the same time, the dominant bacterial genera are *Trichoderma* and *Talaromyces*, each accounting for 40% of the total [34]. In 2017, *Penicillium skriabinii* was isolated from roots collected from Xiaocaoba, Zhaotong, Yunnan [35].

Duan et al. collected samples from healthy Tianma without pests or diseases from five different regions, including wild areas in Zhaotong and Diqing, Yunnan Province; cultivated fields in Zhaotong, Yunnan Province; and wild areas in Yaopingtou and Baiyanlan, Sichuan Province. Tuberous rhizomes and floral stalks of Tianma were sampled at each sampling site for endophytic fungal isolation. A total of 101 fungal strains were recovered and taxonomically identified, which were assigned to 32 distinct genera. Specifically, 69 strains originating from rhizome tissues fell into 26 genera, whereas 32 strains isolated from floral stalks belonged to 12 genera. Genera including *Penicillium*, *Trichoderma*, *Fusarium*, and *Cladosporium* constituted the dominant endophytic fungal taxa associated with *G. elata* in the present investigation. Notably, rhizome tissues of *G. elata* collected from Zhaotong yielded the highest fungal isolation rate, implying that rhizome tissues harbor a higher colonization frequency of endophytic fungal communities. Taxonomic profiling of the 101 endophytic strains derived from the five geographically separated cultivation habitats revealed low inter-site species similarity. This finding demonstrates a broad geographic distribution of fungal taxa and pronounced endophytic diversity shaped by tissue types and growing locations [36].

Three endophytic strains were isolated from fresh and healthy biennial *G. elata*, which were classified as *Herbaspirillum*, *Serratia plymuthica*, and *Acremonium* [37]. Jing et al. also reported high-throughput sequencing results from healthy Tianma samples from different locations, showing that Basidiomycota was the dominant phylum, with *Mycena*, *Thanatephorus* and *Cladophialophora* as the predominant genera [38]. Four endophytic bacteria strains were isolated from fresh *G. elata* samples collected from the Qinba area, including two strains of *Bacillus velezensis*, one strain of *Lelliottia* sp. and one strain of *Rahnella aquatilis* [39]. Endophytic fungi were isolated from tubers of biennial *G. elata* collected from Yingjing County, Sichuan Province, using potato dextrose agar (PDA) medium. A total of 9 endophytic fungi strains were obtained, including three strains of *Fusarium.* sp., five strains of *Neonectria.* sp., and one strain of *Oidiodendron.* sp. At the same time, high-throughput sequencing was performed to evaluate the diversity of endophytic fungal communities associated with *G. elata*. Sequencing results showed that the isolated endophytic fungi were classified into four phyla: *Basidiomycota*, *Ascomycota*, *Zygomycota*, and *Glomeromycota*. Among them, *Ascomycetes* and *Basidiomycetes* constituted the predominant fungal taxa, collectively accounting for 91.52% of the total endophytic fungal community [40]. Two endophytic fungal strains, *Phoma* sp. YUD17001 and *Epicoccum* sp. YUD17002, were isolated from underground tuber tissue of *G. elata* collected from the Xiaocaoba artificial forest in Zhaotong, Yunnan Province [41]. A novel *Phlebiopsis* sp. was also identified from the tubers of *G. elata* in a previous study [42]. In addition, *Cellulomonas endophytica* SYSUP0004T, a facultatively anaerobic and non-motile strain, was isolated from the tubers of *G. elata* Blume collected from Yunnan Province [43]. Moreover, *Sphingomonas* SYSUP0001T—which is Gram-stain-negative, strictly aerobic, and non-motile—was isolated from tubers of *G. elata* Blume [44]. A novel species of the genus *Paracoccus* isolated from *G. elata* was proposed as a new taxon based on its phenotypic, chemotaxonomic, and molecular characterizations, with the Gram-stain-negative strain SYSUP0001T isolated from tubers of *G. elata* Blume [45]. Furthermore, a Gram-stain-positive and nonmotile strain was designated *Amycolatopsis alkalitolerans* sp. isolated from tubers of *G. elata* Blume [46]. Fungal resources associated with *G. elata* have also been continuously explored. *Talaromyces* sp. YUD18002 was isolated from healthy fresh rhizomes of *G. elata* in Zhaotong, Yunnan [47]. A strain of *Aspergillus cristatus* was isolated from stems in Xiaocaoba, Yiliang, Zhaotong, Yunnan [48]. Illumina high-throughput sequencing has been applied to investigate fungal community dynamics throughout the developmental stages of *G. elata*. The results demonstrated that Basidiomycota serves as the predominant fungal phylum during *G. elata* growth, and the relative abundance and diversity of endophytic fungi vary substantially across different developmental stages [49]. Regional heterogeneity significantly shapes the endophytic fungal assemblages of *G. elata*. Distinct quantitative differences in endophytic fungi were observed in *G. elata* populations from Panhe and Yiliang of Zhaotong. Specifically, *Trichoderma* and *Cadophora* dominated the endophytic fungal community of *G. elata* in Panhe, whereas the genus *Cryptocoryne* constituted the dominant fungal group in Yiliang populations. Such spatial differences in the composition and distribution of *G. elata* endophytic fungal communities are potentially attributable to multiple environmental factors, including altitude, illumination condition, vegetation type, tissue developmental stage, annual average temperature, rhizosphere soil properties, and regional climate characteristics [50].

Previous studies have revealed that approximately 17 endophytic fungi, including about five putative new spaces, were characterized from *G. elata*—specifically, *Aspergillus* sp. T2-19 was isolated and identified from *G. elata* samples collected in Zhaotong, Yunnan [51]. In another investigation, a total of 14 endophytic fungi strains were obtained from *G. elata*, ten of which were taxonomically classified into five genera: *Neonectria*, *Ascomycota*, *Fusarium*, *Oidiodendron* and *Dothiorella* [52]. Moreover, twelve endophytic fungal strains were isolated from wild *G. elata* native to Xiaocaoba, Yiliang County, Zhaotong, Yunnan. Among these isolates, only one—an endophytic fungus with the abundance of metabolites—was selected and identified as *Arcopilus* sp. YUD20001 [53]. Additionally, the actinomycete *Streptomyces coelescens* was isolated from *G. elata* samples in Zhaotong [54]. Tang et al. identified an endophytic fungus, *Alternaria* sp. H-35 from Tianma specimens [55]. Other reported fungal isolates from *G. elata* include *Penicillium* sp. YUD17006 [56]. *Trichoderma reesei* was isolated from the plant sample *G. elata* Blume, which was collected from Yingshan County, Hubei Province [57]. A total of 28 endophytic strains were previously isolated from *G. elata* tubers, including 19 fungal strains and 9 bacteria strains [58].

Notably, multiple studies have obtained unidentified endophytic fungal isolates from *G. elata* tissues. Twenty-four endophytic fungal strains were isolated from germinated *G. elata* protocorms, yet these strains have not been fully identified and are currently labeled with temporary codes [59,60]. Furthermore, ten endophytic fungal strains derived from white-stage *G. elata* in Xiaocaoba (Zhaotong, Yunnan Province) remain taxonomically uncharacterized [61]. Similarly, ten endophytic fungal strains isolated from *G. elata* tubers have not been species identified at the species level [62].

This specialization of host–endophyte associations that varies according to tissue type is far from rare, with supporting evidence found in other plant genera. In reality, endophytes frequently show tissue specificity, yet most merely demonstrate a partiality toward certain tissues [63]. The underlying cause may be endophytes’ strong tendency to colonize specific host tissues with unique chemical properties or structural textures [64].

Overall, the diversity and community composition of endophytic fungi in *G. elata* are jointly determined by geographical origin and tissue type. Ascomycota and Basidiomycota dominate the fungal community, with *Penicillium*, *Trichoderma*, *Aspergillus* and *Ilyonectria* being the prevalent core genera across production areas. Tubers are enriched with *Penicillium* and *Epicoccum* species that exhibit high metabolite yields. Significant inter-regional community differentiation indicates that local soil and climate conditions shape distinct endophytic fungal communities. Among all recorded habitats, the Zhaotong *G. elata* population harbors the richest culturable fungal resources.

**Table 1:** Diversity of endophytes from different tissues of *G. elata*.

Origins of Isolated	Microbial Genus	Host	Refs.
Seed	*Schizophyllum* */* *Irpex*	Zhaotong, Yunnan	[29,30]
	*Alternaria*/*Nectria*/*Pseudocercospora*	Hanzhong, Shaanxi and Zhaotong, Yunnan	[31]
	*Trichoderma*/*Alternaria*/*Fusarium*/*Mammularia*/*Arcopilus*/*Podila*/*Mycopan*/*Trametes*/*Stagonosporopsis*/*Aspergillus*/*Ceratobasidium*/*Galactomyces*/*Mucor*/*Cladosporium*/*Schizophyllum*/*Hypomyces*/*Calycina*/*Nigrospora*/*Penicillium*/*Chaetomium*/*Humicola*/*Ovatospora*/*Corynascus*/*Scedosporium*/*Sclerotinia*/*Martirella*/*Pestalotiopsis*/*Talaromyces*/*Clonostachys*/*Ilyonectria*/*Pezicula*/*Epicoccum*/*Botrytis*/*Diaporthe*/*Pseudohumicola*/*Dichotomopilus*/*Linnemannia*/*Rhizopus*/*Lepidodophoron*/*Irpex*/*Exophiala*/*Neopestalotiopsis*	Lianfeng, Yunnan	[33]
Root	*Trichoderma*/*Talaromyces*/*Cryptosporiopsis*	Changbai Mountain, Jilin	[34]
	*Penicillium*	Xiaocaoba, Yunnan	[35]
Scape	*Phomopsis*/*Fusarium*/*Trichoderma*/*Alternaria*/*Mucor*	Baiyi, Guizhou and Zhaotong, Yunnan	[31,32]
	*Cladosporium*/*Penicillium*/*Trichoderma*/*Galactomyces*/*Zygorhynchus*/*Geotrichum*/*Pestalotiopsis*/*Fusarium*/*Tolypocladium*/*Gliocladium*/*Coniothyrium*/*Eutypa/Leptosphaeria*	Zhaotong, Yunnan, Diqing, Yunnan, Baiyanwan, Sichuan, and Yaopingtou, Sichuan	[36]
Flower	*Fusarium*/*Colletotrichum*/*Conimthyrium*/*Epicoccum*	Baiyi, Guizhou and Zhaotong, Yunnan	[32]
Tuber	*Alternaria*/*Fusarium*/*Colletotrichum/Phomopsis*/*Mucor*/*Nectria*/*Epicoccum*/*Gibberella*/*Leptosphaeria*/*Chaetomium*/*Amillaria*/*Mycena*	Bijie, Guizhou, Dafang, Guizhou, Baiyi, Guizhou, Hanzhong, Shaanxi, Zhaotong, Yunnan, Guangyuan, Sichuan, and Sichuan Yingjing	[32]
	*Aspergillus*	Xiaocaoba, Yunnan	[31,48]
	*Herbaspirillum*/*Serratia* /*Acremonium*	Jingyu, Jilin	[37]
	*Bacillus/Lelliottia*/*Rahnella*	Lueyang, Shaanxi	[39]
	*Fusarium*/*Neonectria*/*Oidodendron*	Yingjing, Sichuan	[40]
	*Cladosporium*/*Arthrinium*/*Nigrospora*/*Hypoxylon*/*Penicillium/Trichoderma*/*Alternaria*/*Mortierella*/*Phoma*/*Stereum*/*Cylindrocladiella*/*Galactomyces*/*Aspergillus*/*Mucor*/*Colletotrichum/Geotrichum*/*Schizophyllum*/*Coprinellus*/*Fusarium*/*Bjerkandera*/*Trametes*/*Diaporthe*/*Didymella*/*Proteus*/*Epicoccum/Leptosphaeria*	Zhaotong, Yunnan, Diqing, Yunnan, Yao Pingtou, Sichuan, and Baiyanwan, Sichuan	[36]
	*Phoma*/*Epicoccum*	Xiaocaoba, Yunnan	[41]
	*Phlebiopsis*	Suining, Hunan	[42]
	*Cellulomonas*/*Sphingomonas*/*Paracoccus*/*Amycolatopsis*	-, Yunnan	[43,44,45,46]
	*Talaromyces*	Zhaotong, Yunnan	[47]
-	*Aspergillus*	Zhaotong, Yunnan	[51]
	*Neonectria*/*Fusarium*/*Oidiodendron*/*Dothiorella*	Yingjing, Sichuan	[52]
	*Arcopilus*	Xiaocaoba, Yunnan	[53]
	*Streptomyces*/*Penicillium*	Zhaotong, Yunnan	[54,56]
	*Trichoderma*	Yingshan, Hubei	[57]
	*Alternaria*/*Pycnoporus*	-	[55,58]

## 3. Activity of Endophytes from *G. elata*

*Herbaspirillum* sp., *Serratia plymuthica*, and *Acremonium* sp. Showed significant inhibitory activities against pathogenic bacteria (*Pseudomonas*, *Candida vartiovaarae*, *Penicillium oxalicum*) isolated from diseased *Astragalus membranaceus* and *Melilotus officinalis*, with inhibition rates ranging from 16.67% to 83.33% [37]. Four endophytic bacterial strains isolated from fresh *G. elata* display distinct antibacterial effects against *Escherichia coli* and *Staphylococcus aureus*. Among them, strain B2 presents the best antibacterial ability, with inhibition zone diameters of 14.33 mm and 26.33 mm against *E. coli* and *S. aureus*, respectively. The antioxidant capacities of the fermentation broth derived from four endophytic bacterial strains was measured via scavenging 1,1-diphenyl-2-picrylhydrazyl (DPPH) and hydroxyl (·OH) free radicals scavenging assays. The results revealed that strains B2 possesses the strongest antioxidant capacity (19.26 U/mL), followed by strains B4 and B1. Additionally, the inhibitory activity of the bacterial fermentation broth on the proliferation of A549 cells and human embryonic kidney HEK239T cells was further measured. The study found that strains B1 and B2 exerted an inhibitory effect against A549 cells, with B1 showing the highest inhibitory rate of 8.34%. In contrast, strains B2, B3, and B4 inhibited the growth of human embryonic kidney cells (HEK293T), among which strain B2 achieved the maximum inhibitory efficiency of 40.40%. Additionally, strains B1, B2, and B4 were capable of scavenging DPPH free radicals, and strain B1 exhibited the most prominent scavenging ability with a rate of 69.13% [39]. The antioxidant capacity and free radical scavenging ability of the mycelium ethanol extract were evaluated and the results showed that GER2 (*Ascomycota*), GER10 (*Neonectria* sp.), and GER6 (*Oidiodendron* sp.) possessed good activity, which was speculated to be closely associated with the phenolic compound accumulation in these endophytes. Notably, only GER6 was detected to contain gastrodin, with a content of 39.2 μg/g. These findings indicate that the *G. elata*-derived endophyte *Oidiodendron* has great potential as a natural antioxidant and as a promising alternative resource for *G. elata* exploitation [52]. In another study, using 2,6-di-tert-butyl-4-methylphenol (BHT) set as the positive control, the total antioxidant activity and DPPH free radical scavenging rate of the ethanol extracts from 10 endophytic fungal strains were measured. The total antioxidant capacities of these strains ranged from 367.61 to 318.66 U/g. Specifically, strain GER6 exhibited excellent antioxidant activity with a half-maximal inhibitory concentration (IC_50_) of 465.42 μg/mL, which can serve as a promising candidate for the development of antioxidant drugs [40,52]. Ten strains of endophytic fungi were isolated from *G. elata,* and their effects on plant pathogenic bacteria were measured. The bioactivity assays showed that two strains possessed strong antifungal activity, while the remaining eight strains showed no detectable antibacterial activity, with *Epicoccum* sp. Identified as one of the active strains [62]. Moreover, *Pycnoporus* sp. Was screened as a high-activity antioxidant endophyte, with ·OH, DPPH, and ABTS cation radical scavenging rates of 62.49%, 62.37%, and 71.26%, respectively [58]. Table 2 summarizes the biological activities of *G. elata* endophytes.

## 4. Effects of Endophytes on the Host *G. elata*

Experimental investigations on the functional effects of endophytic fungi isolated from *G. elata* seed protocorms demonstrated that 12 endophytic fungal strains could provide nutritional support for seed germination, among which the most active one was the *Asteria elegans* [65,66]. Multiple microbial strains isolated and identified from healthy *G. elata* tissues, including *Gastrospirillum*, *Serratia puchengeng* and *Acremonium gastrodia,* exert significant inhibitory effects on the phytopathogen *Penicillium oxalicum* derived from diseased *G. elata* individuals [37]. Some fungal genera recovered from *G. elata* can exhibit dual-sided biological properties.Pathogenicity assays revealed that *Fusarium* and *Pythium* possess strong pathogenicity to *G. elata*, while *Metarhizium*, *Pseudomonas Trichoderma*, *Trichoderma*, and *Polyporus* exhibit moderate pathogenicity. Notably, strains within the genus *Trichoderma* display differential pathogenic characteristics toward *G. elata*. Specifically, *T. viride* and *T. chlamydomonas* show the highest pathogenicity, whereas *T. erconii*, *T. melanogaster*, *T. spiralis*, and *T. sulfa* exhibit no pathogenicity to *G. elata*. Furthermore, this study is the first to report the pathogenicity of *Pythium* species against *G. elata* [38].

## 5. Secondary Metabolites and Biological Activities of Endophytics in *G. elata*

**Figure 2:**
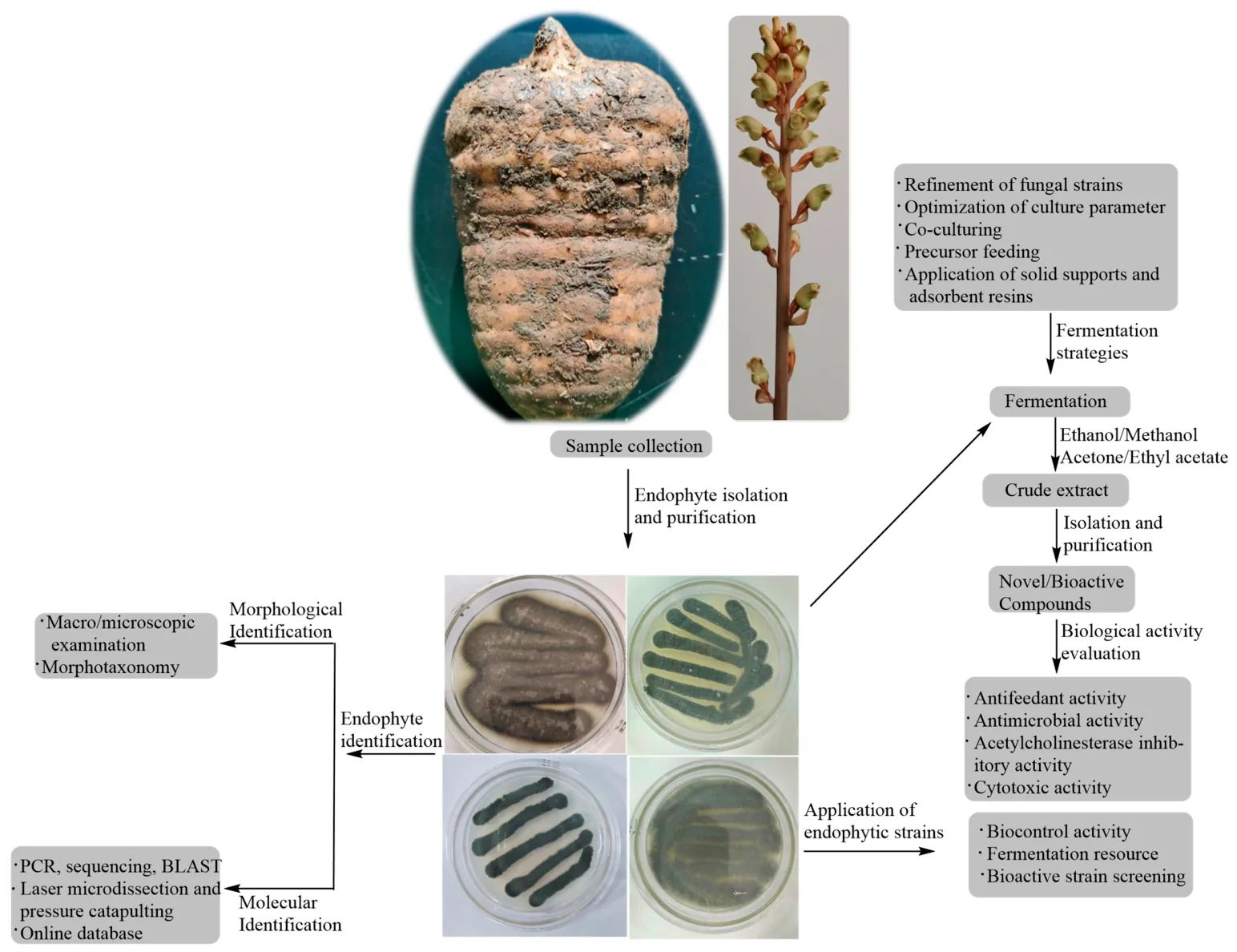
Research workflow for isolation, identification, and application of endophytes from *G. elata*, as well as discovery of their bioactive secondary metabolites.

**Figure 3:**
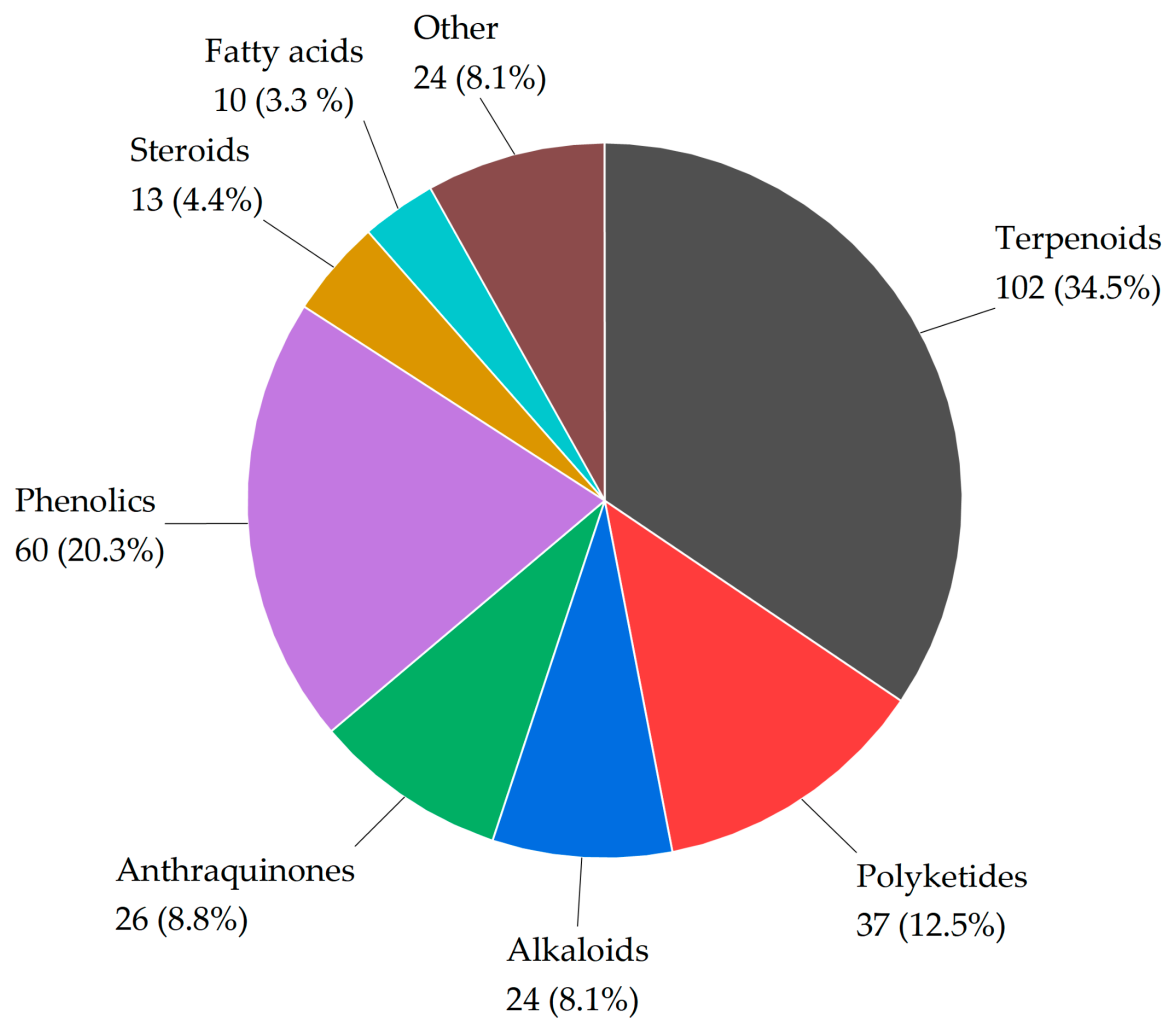
Classification of the secondary metabolites from the *G. elata*-associated endophytes.

Currently, research on the secondary metabolites of *G. elata* endophytes are mainly focused on endophytic fungi, while studies concerning endophytic bacteria and actinomycetes. The stepwise procedure for isolation of bioactive metabolites from fungal endophytes and their biological applications is presented in Figure 2. Endophytic fungi of *G. elata* yield abundant secondary metabolites—including terpenoids, polyketides, alkaloids, steroids, phenolics, fatty acids, and other compounds—and its major metabolites are classified as terpenoids, polyketides, and fatty acid. The present review describes 296 chemical constituents, which are mainly divided into terpenoids (102), polyketides (37), alkaloids (24), anthraquinones (26), phenols (60), steroids (13), fatty acids (10), and other compounds (24), as shown in Figure 3.

### 5.1. Terpenoids

**Figure 4:**
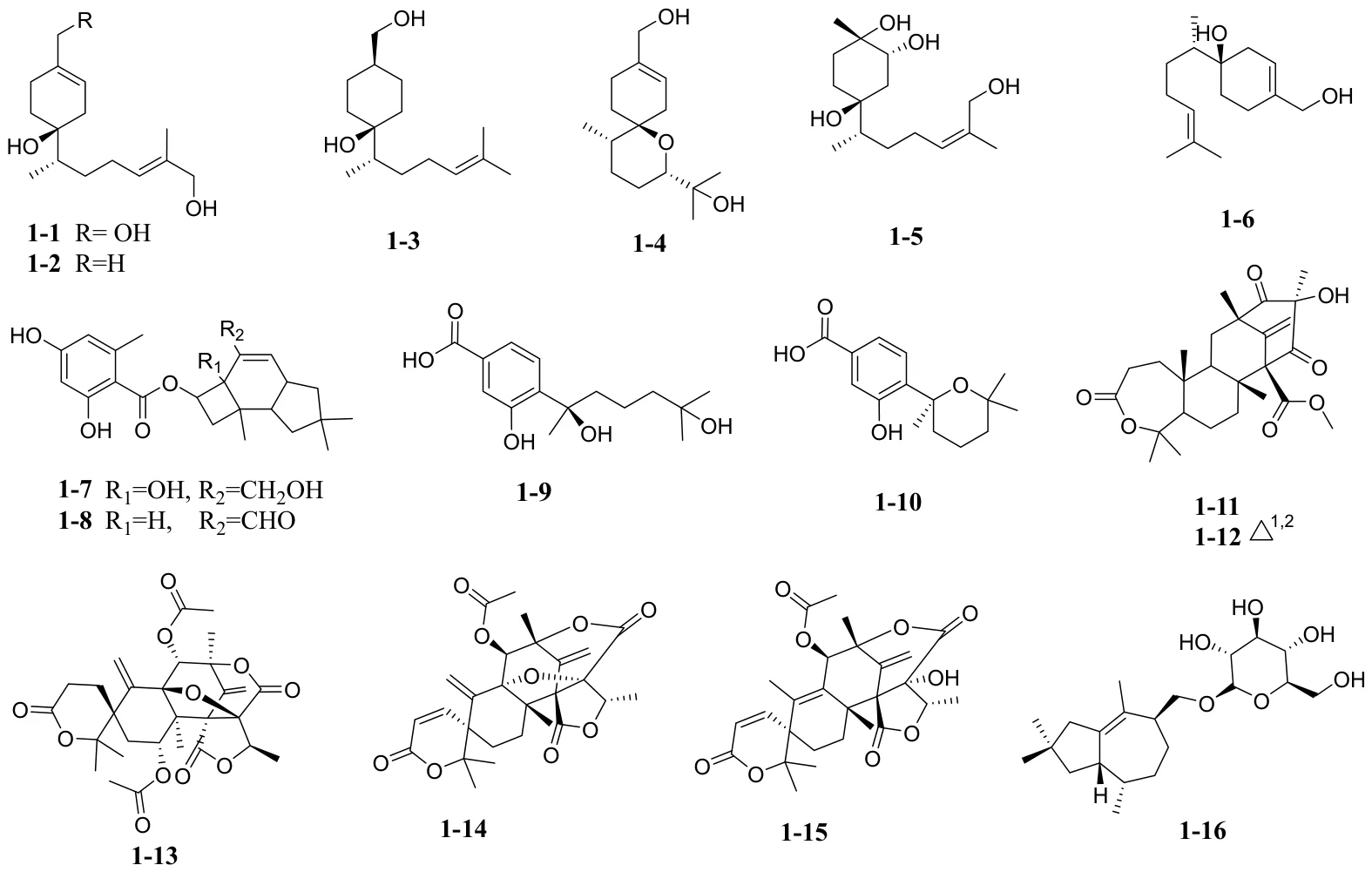

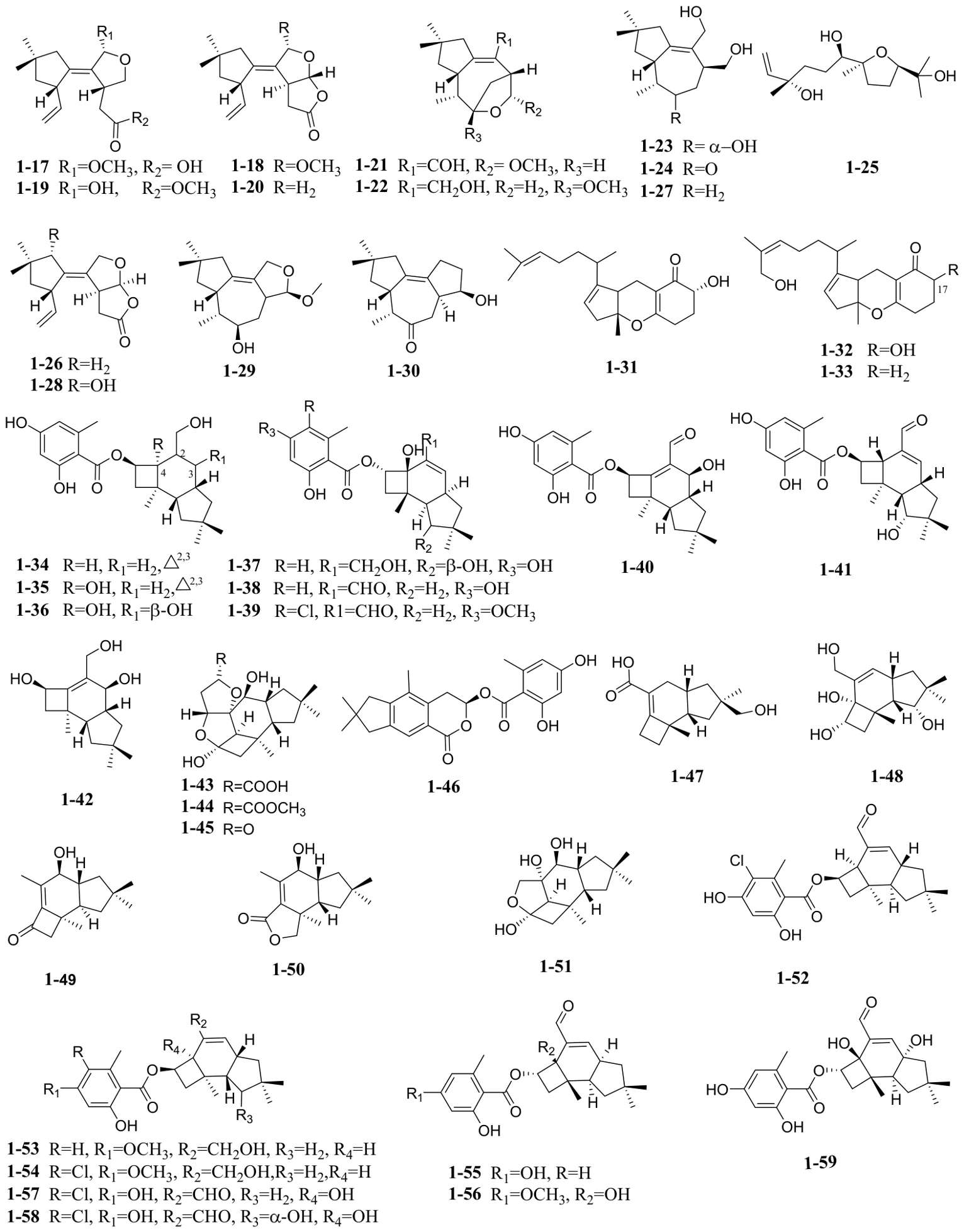

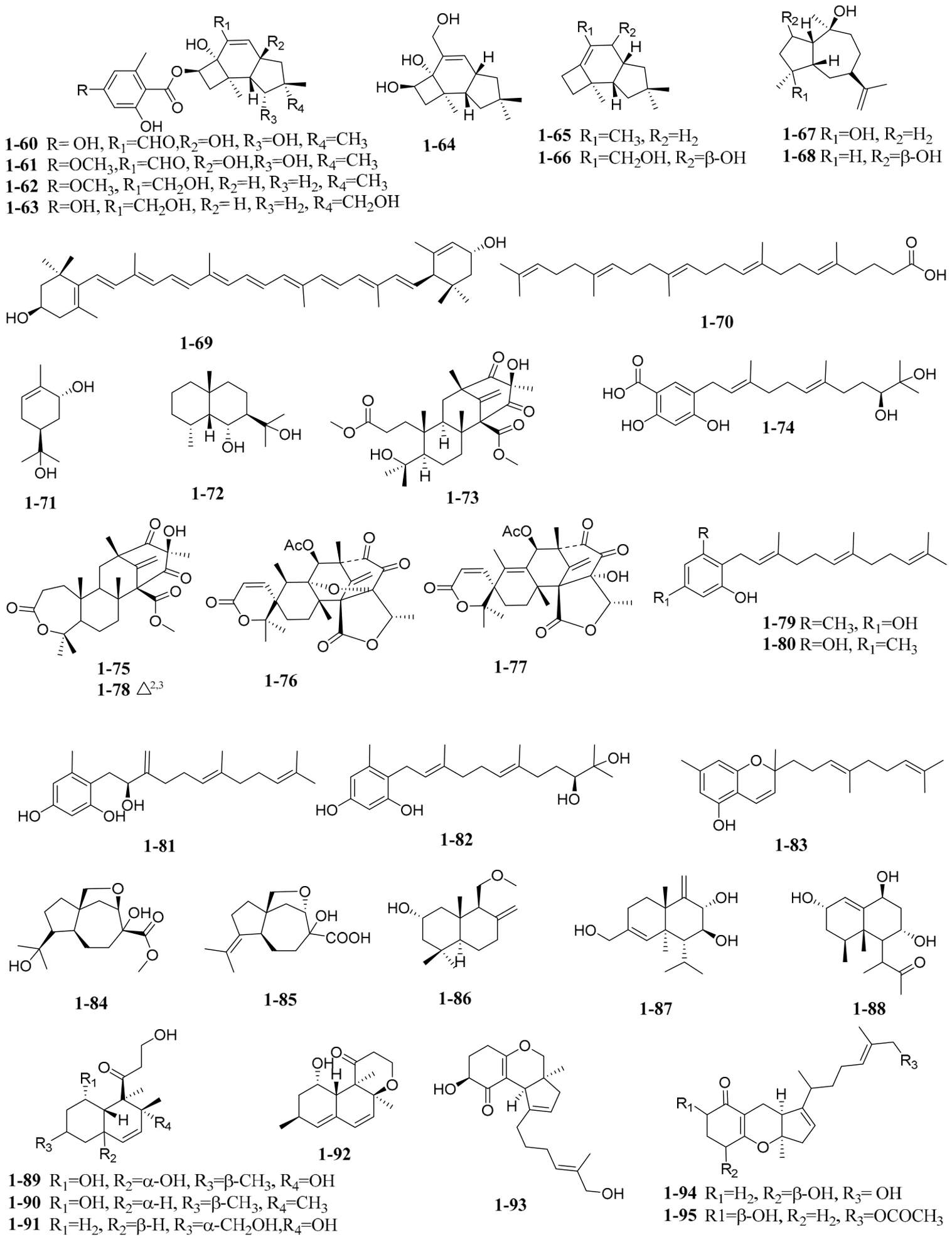

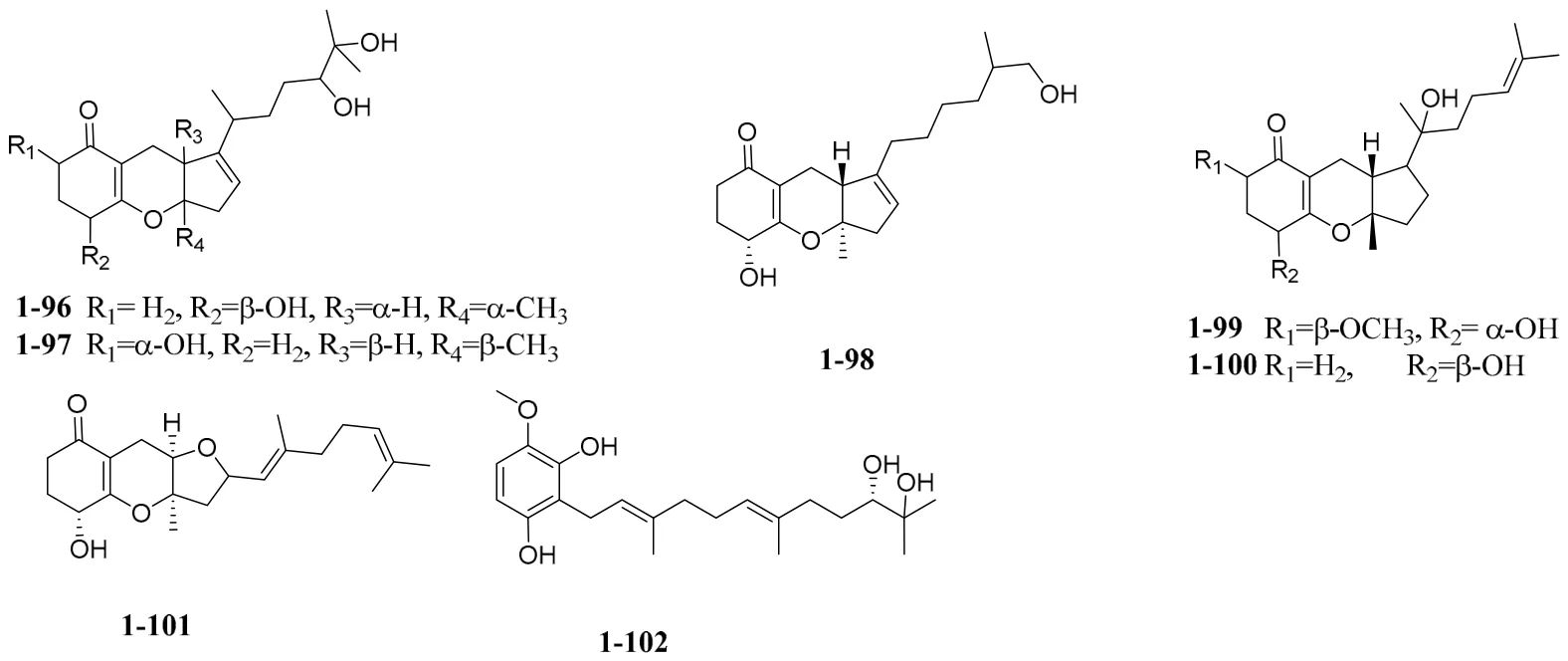
Terpenoids **1-1**–**1-102** in endophytic fungi of *G. elata*.

To date, terpenoids account for the highest proportion among the compounds isolated and identified from endophytic fungi of *G. elata*, with a total of 102 monomeric compounds, which are summarized in Figure 4 and Table 3. Most terpenoids with potent antitumor and acetylcholinesterase (AchE) inhibitory activity are recovered from co-cultures with *Armillaria mellea*. Taxonomic bias is evident in terpenoid biosynthesis: basidiomycetes including *Schizophyllum* and *Irpex* predominantly generate sesquiterpenes bearing antifeedant properties, whereas ascomycetous *Penicillium* and *Aspergillus* produce structurally diverse antibacterial terpenoid derivatives.

Six sesquiterpenes (**1-1**–**1-6**) were isolated from the endophytic fungus *Schizophyllum commune* from Zhaotong *G. elata*, including five new bisabolane sesquiterpenes (**1-1**–**1-5**) along with one structurally related known compound (**1-6**). In addition, all compounds showed antifeedant activity against silkworms, with feeding deterrence indices (FDIs) ranging from 21% to 85%. Compounds **1-1**, **1-3**, **1-4**, and **1-5** showed synergistic insecticidal effects when combined with abamectin, stronger than abamectin alone [29]. 4-Dehydrodihy-dromelIeolide (**1-7**) and armillarivin (**1-8**), two sesquiterpenoids, were identified from the co-culture of *Aspergillus* sp. T2-19 and *Armillaria* sp. in PDB medium. Subsequently, the culture medium was changed to study the secondary metabolites of the solid co-culture of these two fungi, from which two bisabolane-type terpenoids (**1-9** and **1-10**) were discovered. Compound **1-10** showed no inhibitory activity against HL-60 cells at a concentration of 100 μg/mL [51]. The secondary metabolites of the endophytic fungus *Penicillium skriabinii* were investigated using solid-state fermentation, and five terpenoids (**1-11**–**1-15**) were isolated. Bioactivity studies revealed that compound **1-14** exhibited inhibitory activity against acetylcholinesterase with an IC_50_ value of 0.40 μM [35]. Fifteen terpenoids (**1-16**–**1-30**), including five new sesquiterpenoids (**1-16**–**1-20**), were isolated from the mixed PDB medium of the endophytic fungus *I. lacteus*. Compounds **1-16** and **1-22** exhibited significant antifungal activity against *Penicillium polonicum*, and compounds **1-17**, **1-21** and **1-25** against *Psathyrella subsingeri*, wth MICs of 8 μg/mL [30,67]. The endophytic fungus *Alternaria* sp. H-35 was subjected to large-scale fermentation using PSD medium, and the fermentation products were isolated to yield meroterpenoids (**1-31**–**1-33**), among which compound **1-31** exhibited weak antimicrobial activity against *Staphylococcus aureus*, *Escherichia coli* and *Candida albicans*, with MIC values of 128–256 μg/mL [55]. Li et al. studied the co-culture system of *Phoma* sp. YUD17001 isolated from *G. elata* and *Armillaria* sp. A systematic chemical study of the secondary metabolites from the co-culture led to the isolation of nine sesquiterpenoids (**1-34**–**1-42**). At a concentration of 40 μM, compound **1-34** showed strong inhibitory activity against five human tumor cell lines (colorectal cancer SW480, breast cancer MCF-7, liver cancer SMMC-7721, leukemia HL-60, and lung cancer A-549), with IC_50_ values of 13.43 ± 0.58, 17.92 ± 0.74, 15.98 ± 0.99, 16.59 ± 0.16, and 17.32 ± 0.37 μM, respectively. The inhibitory activity against MCF-7 and SW480 was stronger than that of cisplatin. At a concentration of 50 μM, compound **1-34** also showed AChE inhibitory activity, with an IC_50_ value of 19.03 ± 0.28 μM, and moderate inhibitory activity against *Staphylococcus aureus* and *Bacillus subtilis* [41]. Twenty-nine terpenoids (**1-43**–**1-71**)—including illudalane-type sesquiterpene derivatives, protoilludane sesquiterids, and monoterpenoids—were isolated from the metabolites of co-cultured endophytic fungus *Epicocum* sp. YUD17002 and *Armillaria mellea*. Activity testing revealed that compound **1-46** exhibited strong acetylcholinesterase inhibitory activity with an IC_50_ value of 4.91 μM. Compound **1-52** showed moderate in vitro cytotoxic inhibitory activity against five human tumor cell lines (SW480, 49, MCF-7, SMMC-7721, and HL-60) with IC_50_ values ranging from 15.80 to 3.03 μM and also demonstrated moderate acetylcholinesterase inhibitory activity. Additionally, compounds **1-46**, **1-47**, **1-49**, **1-50**, and **1-52** exhibited good antibacterial activity [41,68,69]. The secondary metabolites of the endophytic fungus *Penicillium* sp. TG-46 were systematically investigated, yielding one terpenoid compound (**1-72**) [41]. *Penicillium* sp. T2-8, an endophytic fungus isolated from the tuber tissue of *G. elata*, was investigated for its secondary metabolites, leading to the isolation of 11 terpenoids (**1-73**–**1-83**). Notably, Preaustinoid A1 (**1-75**) showed strong antibacterial activity against *Bacillus subtilis*, with an MIC value of 4 μg/mL that was equivalent to that of the reference antibiotic Kanamycin [36,70]. Neogrifolin (**1-79**) likewise showed strong inhibitory activity against *E. coli* and *S. aureus*, with respective MICs of 8 and 4 μg/mL, consistent with kanamycin. Further cytotoxicity screening across five malignant cell lines (HL-60, A-549, SMMC-7721, MCF-7, and SW480) indicated that compounds **1-73** and **1-74** lacked obvious cytotoxic potential, as their IC_50_ values were all higher than 40 μM [36]. *Enicillium* sp. T2-8, an endophytic fungus of *G. elata*, was subjected to combined mutagenesis using ultraviolet light (UV, λ = 360 nm) and the chemical mutagen nitrosoguanidine (NTG, 1-methyl-3-nio-1-nitrosoguanidine), resulting in the screening of the mutant strain *Penicillium* sp. T2-M10. The metabolic products of this strain were investigated, and two terpenoid compounds (**1-84** and **1-85**) were isolated. Among them, compound **1-84** exhibited inhibitory activity against the growth of three bacterial strains (*B. subtilis*, *E. coli*, and *S. aureus*), with MIC values of 8, 8, and 4 μg/ML, respectively. The metabolites of the endophytic fungus *Penicillium* sp. T2-11 were studied, and three terpenoids (**1-86**–**1-88**) were discovered. The investigation of the metabolites from endophyte *Aspergillus* sp. T2-19 cultured in host “Tianma” revealed four terpenoids (**1-89**–**1-92**). Ten terpenoid compounds (**1-93**–**1-102**) were isolated from the secondary metabolites of the endophytic fungus *Alternaria* TY2-3, and the antibacterial activity tests showed that none of the compounds exhibited good antibacterial activity [36].

### 5.2. Polyketides

**Figure 5:**
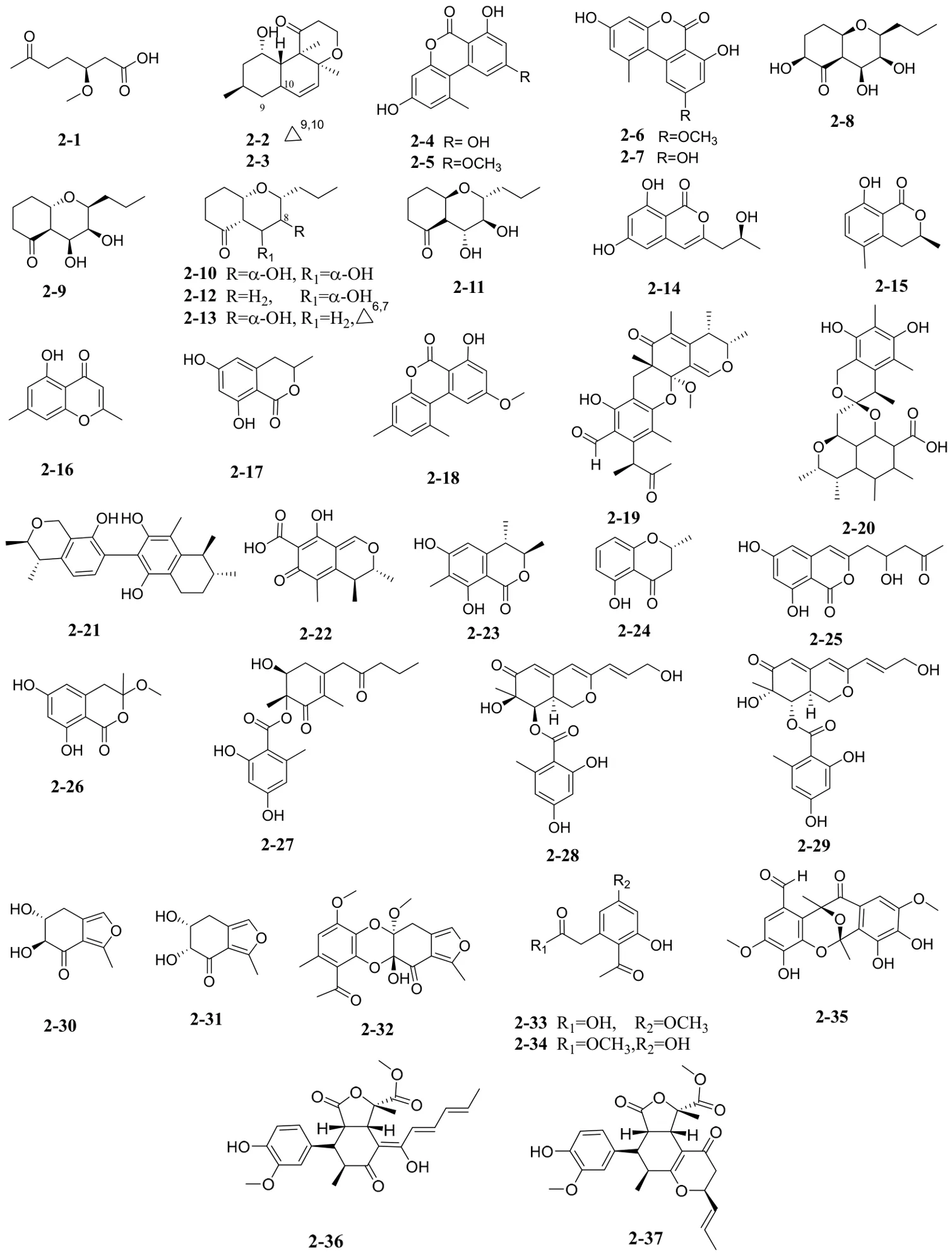
Polyketides **2-1**–**2-37** in endophytic fungi of *G. elata.*

Polyketides are a large family of natural products that are known to exhibit a high degree of structural diversity with a wide range of biological activities [71,72,73,74,75,76,77,78,79,80]. Plants, bacteria, and fungi can produce polyketides through polyketide synthase enzymes [81,82]. Polyketides of fungal origin have diverse structures, ranging from antibiotics to toxins, which are widely used in pharmacological aspects [83,84]. The polyketide metabolites and their biological activities are shown in Figure 5 and Table 4. One new polyketide compound (**2-1**) was isolated from the endophytic fungus *Schizophyllum commune* of *G. elata* from Zhaotong, which exhibited antifeedant activity against the silkworms, and also possessed significant insecticidal activity with a mortality rate of 60% [29]. Versiol (**2-2**) and Isoversiol (**2-3**), two polyketide compounds, were isolated from the fermentation broth of PDB containing the endophytic fungus *Aspergillus* sp. T2-19 and *Armillaria mellea* [51]. Taking the endophytic fungus *Arcopilus* sp. YUD20001 as the research object, its secondary metabolites were studied via solid-state fermentation, from which two polyketide compounds (**2-4** and **2-5**) were obtained [53]. Co-culturing the endophytic fungus *Phoma* sp. YUD17001 from *G. elata* with *Armillaria* sp. in liquid nutrient medium resulted in the production of six new secondary metabolites, phomretones A–F (**2-8**–**2-13**), which were a series of structurally related C12 polyketides that differ in the geometry and substitution pattern, and three known polyketide compounds (**2-14**–**2-16**) [41]. The in vitro cytotoxic inhibitory activities of compounds **2-8**–**2-13** against five human cancer cell lines, including hepatocellular carcinoma (SMMC-7721), lung cancer (A-549), leukemia (HL-60), breast cancer (MCF-7), and colorectal cancer (SW480), were evaluated using the MTS assay. The preliminary screening results showed that those compounds exhibited no inhibitory activity against them at a concentration of 40 μM [78]. In addition, their acetylcholinesterase inhibitory activity and antibacterial activity were also tested, but no activity was observed [41,85]. The chemical investigation of the ethyl acetate (EtOAc) extract from the fermentation medium of the endophytic fungus *Penicillium* sp. T2-8 was carried out, which led to the isolation of a polyketide compound (**2-17**) [36]. A polyketide compound (**2-18**) was isolated from the secondary metabolites of the artificially mutagenized strain *Penicillium* sp. T2-M20, which was subjected to combined mutagenesis using ultraviolet light (UV, λ = 360 nm) and the chemical mutagen nitrosogunidine (NTG, 1-methyl-3-nitro-1-nitrosoguanidine) [36]. The secondary metabolites of the endophytic fungus *Penicillium* sp. T2-11 from *G. elata* were isolated, yielding seven polyketide compounds (**2-19**–**2-25**). Compound **2-19** showed significant antimicrobial effects against *Candida albicans* and *Bacillus subtilis*, sharing the same MIC of 4 μg/mL against the two tested strains. Compound **2-20** also exhibited bioactivity against these two microorganisms, with MIC values of 4 and 8 μg/mL, respectively. Meanwhile, subsequent cytotoxic screening against five human tumor cell lines further uncovered that compound **2-19** displayed varying degrees of inhibitory effects against five human tumor cell lines. Notably, its cytotoxic potency against MCF-7 cells surpassed that of cisplatin, whereas its inhibitory capacity against SMMC-7721 and A-549 cells was close to the reference drug cisplatin [36]. One polyketide compound (**2-2**) was isolated from the endophytic fungus *Aspergillus* sp. T2-19 of *G. elata*, and two polyketide compounds (**2-5** and **2-26**) were isolated from the secondary metabolites of the endophytic fungus *Alternaria* sp. TY2-3 [36]. Chemical analysis and isolation of the *G. elata*-derived endophytic fungus *Talaromyces* sp. YUD18002 secondary metabolites, led to the discovery of a novel azaphilone analog, talarophilone (**2-27**), together with two known compounds—epi-pinophilin B (**2-28**) and pinophilin B (**2-29**). The in vitro cytotoxicities of **2-27** against human myeloid leukemia HL-60, human hepatocellular carcinoma SMMC-7721, lung cancer A-549, breast cancer MCF-7 and human colon cancer SW480 were assayed using MTS method, assayed using MTS method, and compound **2-27** had no obvious cytotoxicity (IC_50_ > 20 uM) to the five cells [47]. Three novel, highly oxygenated polyketides, multioketides A–C (**2-30**–**2-32**), and three previously described multioxidized aromatic polyketides (**2-33**–**2-35**), were isolated from an endophytic *Penicillium* sp. YUD17006 associated with *G. elata.* Additionally, compound **2-32** demonstrated weak inhibitory activities against both acetylcholinesterase and protein tyrosine phosphatase 1B [56]. Trichodenoids A (**2-36**) and B (**2-37**), two skeletally unprecedented polyketides, were isolated from the endophytic fungus *T. reesei* originating from the plant *G. elata* Blume. Moreover, the biological effects showed that compound **2-37** had the potential to mitigate H_2_O_2_-induced oxidative stress in H9C2 cells by reducing intracellular ROS levels, restoring mitochondrial function, and regulating the mRNA expression related to oxidative stress, inflammation, and autophagy. These findings highlight compound **2-37** as a potential candidate for natural antioxidants and even as dietary supplements for cardiovascular health [57].

Polyketides are widely distributed in ascomycetous endophytes. Many strains of *Penicillium* can generate polyketides with broad spectrum bacteriostasis, yet most polyketides lack potent cytotoxic activity against tumor cells compared with terpenoids.

### 5.3. Alkaloids

**Figure 6:**
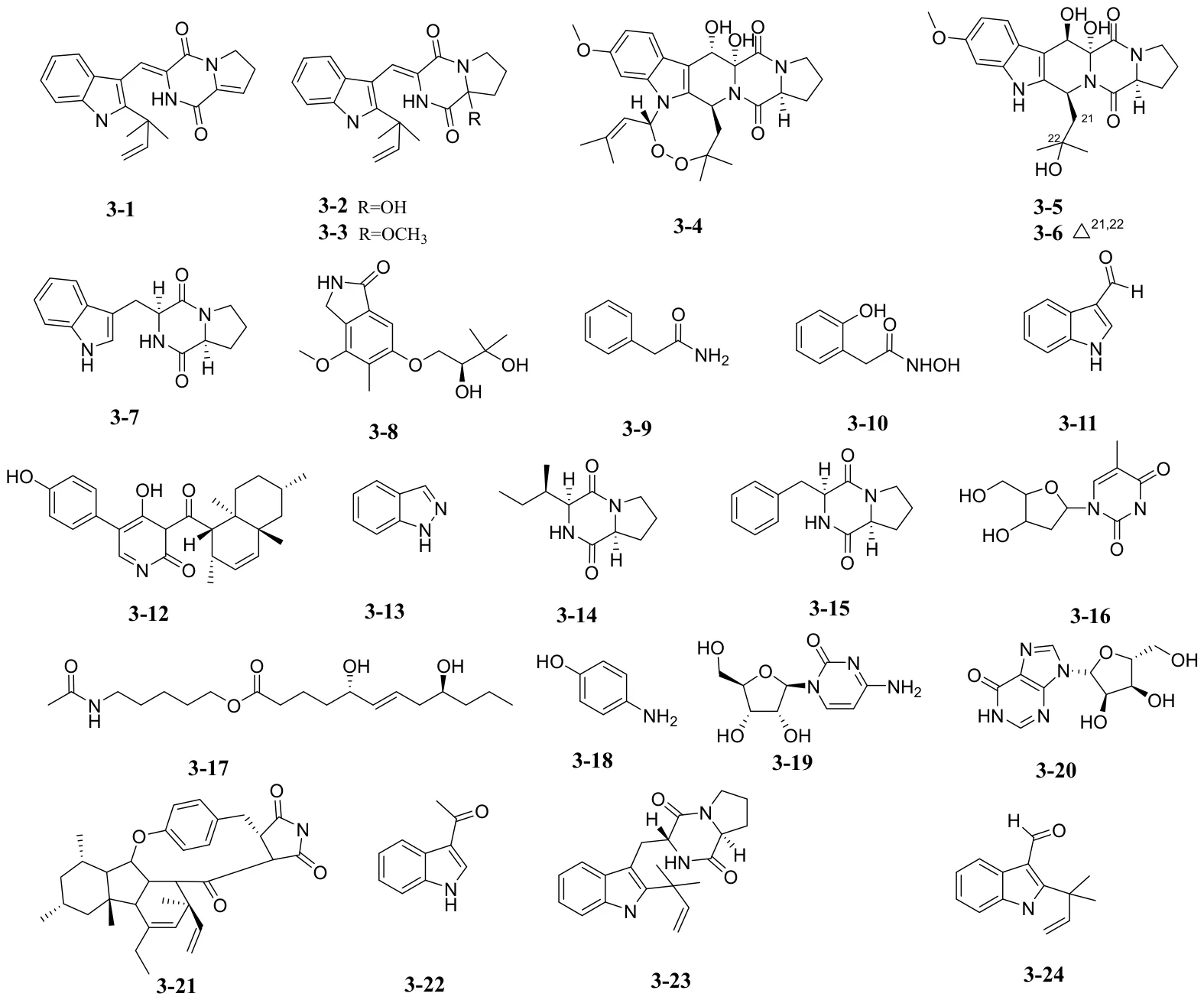
Alkaloids **3-1**–**3-24** in endophytic fungi of *G. elata*.

Alkaloids are prevalent bioactive secondary metabolites found in plants, as well as an important group of metabolites produced by their endophytes [86,87,88,89,90,91]. A total of twenty-four alkaloid compounds have been isolated and identified from the endophytic fungi of *G. elata,* with their structures shown in Figure 6 and Table 5. Among them, three diketopiperazine alkaloids (**3-1**–**3-3**) were obtained from the solid co-culture of the *Armillaria* sp. and the endophytic fungus *Aspergillus* sp. T2-19 associated with *G. elata*. Compounds **3-1** and **3-3** showed inhibitory activity against leukemia HL-60 cells at a concentration of 100 μg/mL, with inhibition rates of 97.60 ± 0.94% and 97.06 ± 0.15%, and IC_50_ values of 41.16 ± 0.8 and 36.66 ± 1.07 μg/mL, respectively. Additionally, compound **3-1** exhibited antibacterial activity against *Staphylococcus aureus* with a MIC of 256 μg/mL [51]. Four alkaloid compounds (**3-4**–**3-7**) were reported from the secondary metabolites of the endophytic fungus *Penicillium skriabinii*. In addition, compounds **3-4** and **3-6** showed inhibitory activity against *Bacillus subtilis*, *Staphylococcus aureus*, *Escherichia coli*, and *Salmonella*. Verruculogen (**3-4**) exhibited antifeedant activity against armyworms, with an antifeedant index of 55% [35]. A new compound, Arcopiniols C (**3-8**), along with five previously indentified compounds (**3-9**–**3-13**), was isolated from the endophytic fungus *Arcopilus* sp. YUD20001 associated with *G. elata* using various chromatographic separation techniques [53,92]. The isolation of two alkaloids (**3-14** and **3-15**) from the endophytic fungus *Streptomyces coelescens* was carried out [54]. The strain *Alternaria* sp. H-35, an endophytic fungus, was subjected to large-scale fermentation using PDB medium, and one alkaloid (**3-16**) was isolated from it [55]. Two alkaloids (**3-17** and **3-18**) were isolated and identified from the co-culture of the endophytic fungus *Phoma* sp. YUD17001 with *Armillaria mellea*, and one alkaloid (**3-19**) was isolated and identified from the co-culture of *Epicoccum* sp. YUD17002 with *Armillaria* sp. [41,93]. After composite mutagenesis of the wild endophytic fungus *Penicillium* sp. T2-8 from *G. elata*, the mutant strain *Penicillium* sp. T2-M10 was obtained, and one alkaloid (**3-20**) was isolated from its metabolites. Two known alkaloid compounds (**3-21** and **3-22**) were isolated from the secondary metabolites of the endophytic fungus *Penillium* sp. T2-11. Epi-deoxybrevianamide E (**3-23**) and 3-carbaldehydes (**3-24**) were isolated from the secondary metabolites of the endophytic fungus *Aspergillus* sp. T2-19 [36].

### 5.4. Anthraquinones

**Figure 7:**
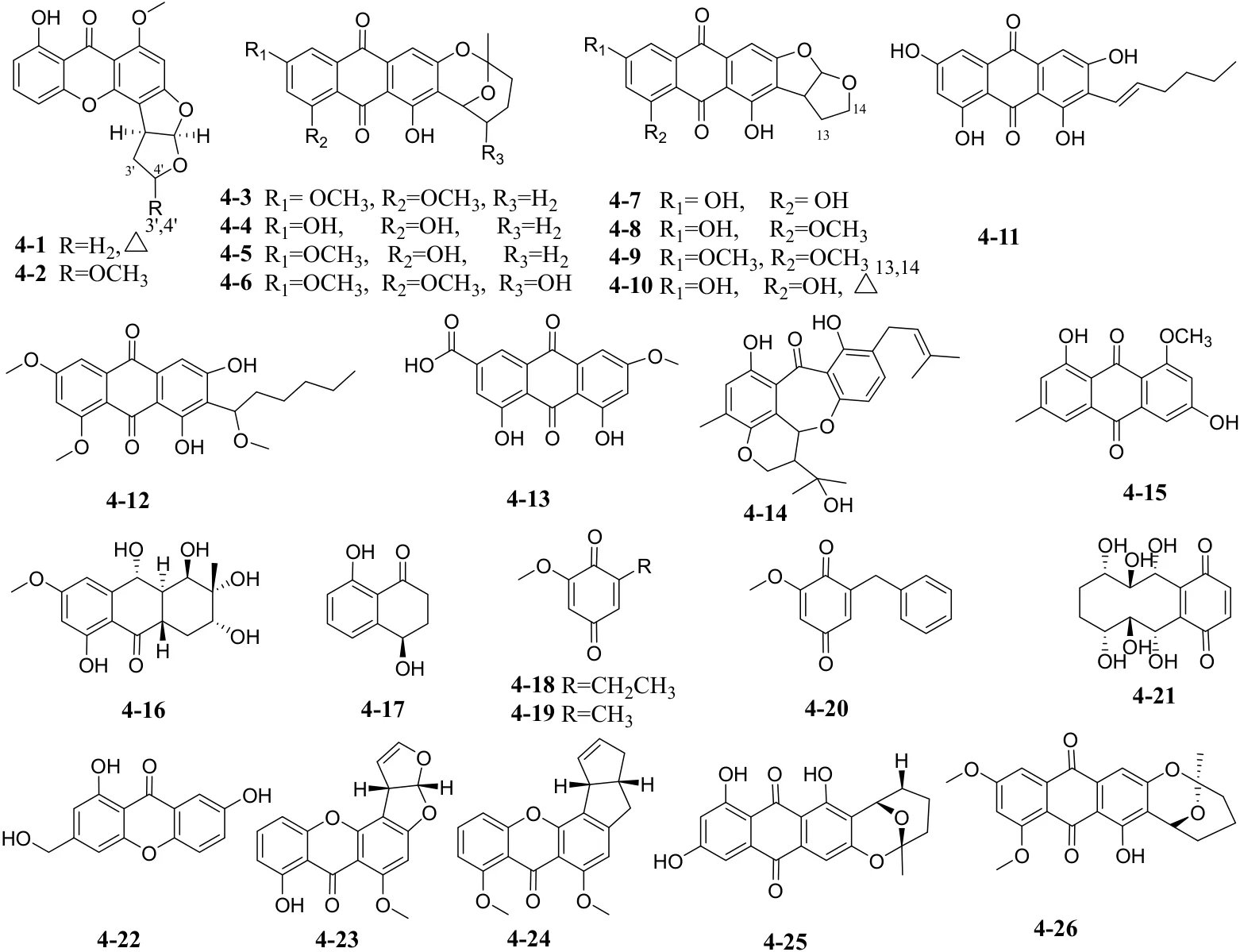
Anthraquinones **4-1**–**4-26** in endophytic fungi of *G. elata*.

As shown in Figure 7 and Table 6, a total of 26 anthraquinones (**4-1**–**4-26**) have been purified from the endophytic fungus associated with *G. elata*. Co-culturing of the endophytic fungus *Aspergillus* sp. T2-19 with *Armillaria* sp. in liquid nutrient medium resulted in the production of 11 anthraquinone compounds (**4-1**–**4-11**); while switching to a rice medium, 6,8,1′-tri-O-methylaverantin (**4-12**), Parietinic acid (**4-13**), and Arugosin C (**4-14**) were obtained from the fermentation products [51]. The antimicrobial activity results showed that compounds **4-7** and **4-9** exhibited good inhibitory activity against *Candida albicans*, with their MIC values being 64 and 32 μg/mL, respectively. Meanwhile, compound **4-7** exhibits inhibitory activity against *Staphylococcus aureus*, with an MIC value of 8 µg/mL, consistent with the positive control kanamycin; Compound **4-6** exhibits inhibitory activity against *Escherichia coli*, with an MIC value of 4 μg/mL. Moreover, 6, 8, 1′-tri-O-methylaverantin (**4-12**) exhibited strong cytotoxic activity against leukemia cells HL-60, with an IC_50_ value of 14.37 μg/mL. Compounds **4-4**, **4-6**, **4-7** and **4-9** also showed cytotoxic effects against this cell line. Questin (**4-15**) was isolated from the fermentation broth of the endophytic fungus *Aspergillus cris* T2-10 from *G. elata* [48]. The isolation of an anthraquinone compound (**4-16**) from the endophytic fungus *Arcopilus* YUD20001 associated with *G. elata* was carried out [53]. A previously reported anthraquinone (**4-17**) was isolated from the co-culture of the endophytic fungus *Phoma* sp. YUD17001 and *Armillaria mellea* [41]. The endophytic fungus *Penicillium* sp. T2-8 isolated from the tuber tissue of *G. elata* was conducted on the chemical constituents of its secondary metabolites, and three anthraquinones (**4-18**–**4-20**) were purified from it. Screening of mutant strains obtained by artificial mutagenesis of the wild strain *Penicillium* sp. T2-8 revealed that the metabolites of the mutant strain *Penicillium* sp. T2-M20 yielded two anthraquinone compounds (**4-19** and **4-21**). The secondary metabolite of the endophytic fungus *Penicillium* sp. T2-11 isolated from *G. elata* yielded huperxanthones B (**4-22**). Averisin (**4-9**), along with four analogs—Sterigmatocystin (**4-23**), O-methylsterigmatocystin (**4-24**), Averufin (**4-25**), and 6,8,1′-tri-O-methylaverantin (**4-26**)—was isolated from the secondary metabolites of the endophytic fungus *Aspergillus* sp. T2-19 [36].

### 5.5. Phenolics

**Figure 8:**
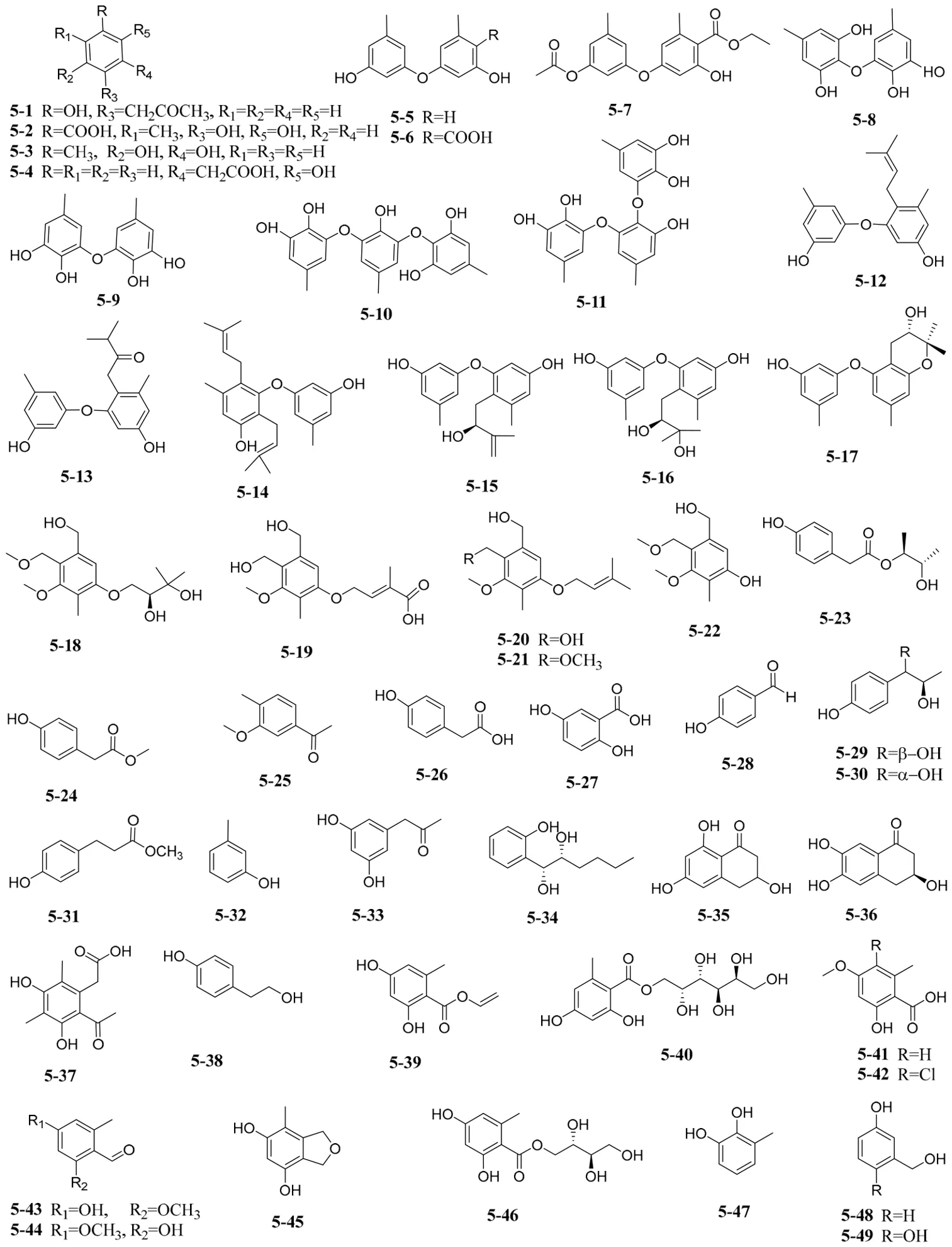

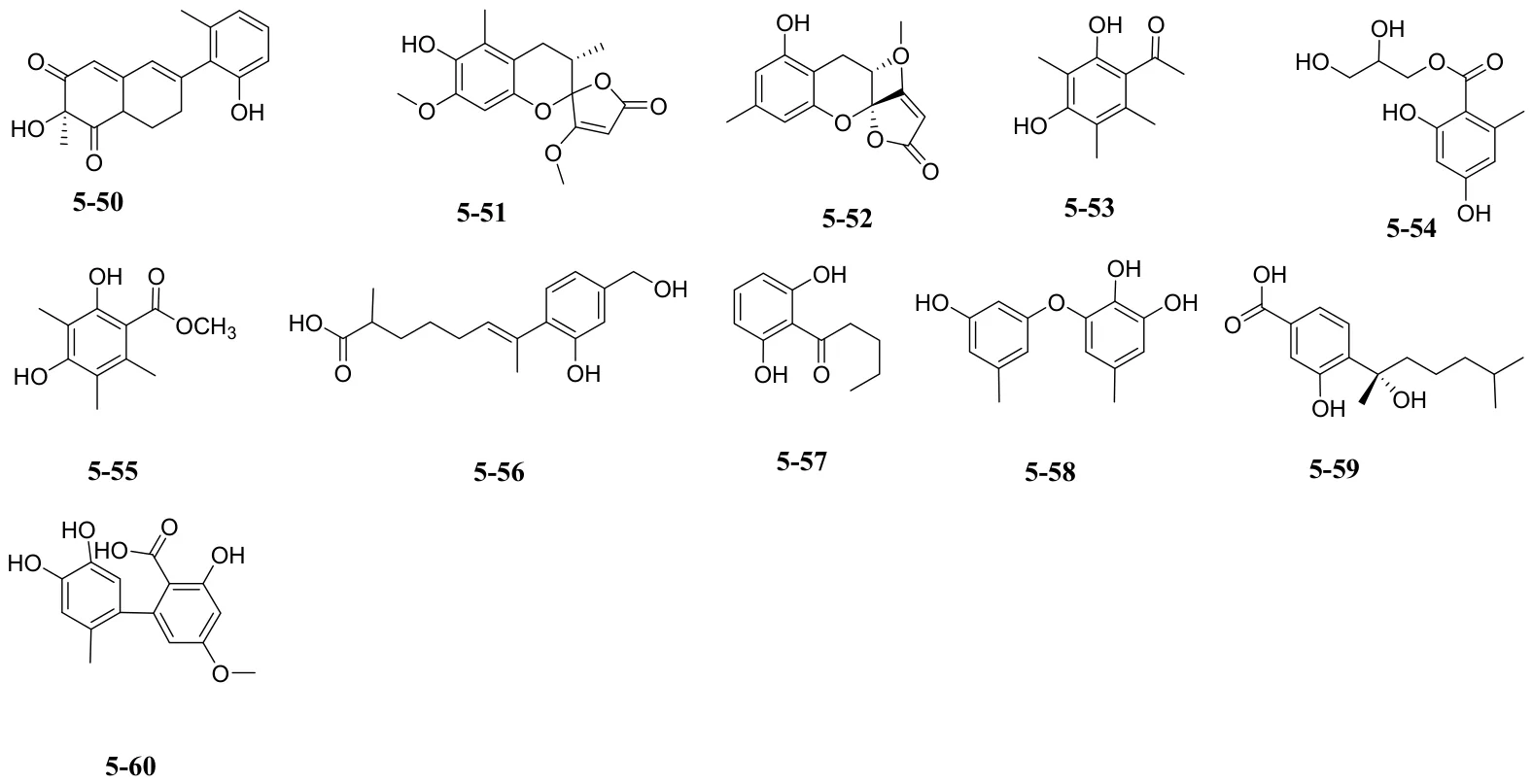
Phenolics **5-1**–**5-60** in endophytic fungi of *G. elata*.

Phenolics constitute the second largest class of secondary metabolites, with 60 congeners and several structural types. These compounds also are ubiquitous secondary metabolites in plants, animals, and microorganisms formed by one or more hydroxyl groups (Methoxy group) replacing hydrogen atoms on the benzene ring of aromatic hydrocarbon [94,95,96]. They are commonly present in fruits, nuts, vegetables, and cereals, which play important roles in attracting pollinators, seed dissemination and plant defense responses [97,98,99,100]. At present, 60 phenolic compounds (**5-1**–**5-60**) have been discovered or isolated from Gastrodia endophytes, namely simple phenols, phenolic glycosides, phenolic ethers, phenolic aldehydes, sulfur-containing phenols, and nitrogen-containing phenols (Figure 8 and Table 7). An endophytic fungus *Schizophyllum commune* was isolated from the seeds of Tianma, and its secondary metabolites were separated, yielding one phenolic compound (**5-1**) [29]. An endophytic fungus *Aspergillus* sp. T2-19 was isolated from *G. elata*, and 10 phenolic compounds (**5-2**–**5-11**) were isolated from the co-fermentation of it with PDB of *Armillaria mellea*. Subsequently, the medium was changed, and six phenolic compounds (**5-12**–**5-17**) were isolated from the secondary metabolites after solid co-cultivation mentation of *Aspergillus* sp. T2-19 with Armillaria. Compound **5-14** exhibited strong cytotoxic activity against leukemia HL-60 tumor cells with an IC_50_ of 8.31 μg/mL. Additionally, Diorcinol E (**5-13**), Benzeneethanol (**5-15**) and (S)-5-(3-hydroxy-5-methylphenoxy)-2,2,7-trimethylchroman-3-ol (**5-17**) also showed cytotoxic activity against leukemia HL-60 tumor cells, with IC_50_ values ranging from 36.42 to 38.71 μg/mL. Compound **5-15** demonstrated antibacterial activity against *Escherichia coli* with an MIC value of 8 μM [51]. Two undescribed zinniol derivatives, arcopiniols A and B (**5-18** and **5-19**), together with seven known compounds (**5-20**–**5-26**), were isolated from the secondary metabolites of the endophytic fungus *Arcopilus* sp. YUD20001 associated with *G. elata* [53]. Further studies demonstrated that arcopiniols A (**5-18**) and B (**5-19**) showed no antitumor activity against tumor cell lines HL-60, A549, SMMC-7721, MDA-MB-231, and SW480 at a concentration of 40 μM. Additionally, compound **5-18** exhibited no inhibitory activity against acetylcholinesterase at 50 μM, while compound **5-19** showed weak activity with an inhibition rate of 16.41% [92]. In addition, phenolics **5-18** and **5-19** had no significant NO inhibitory activity at 50 μg/mL [53]. The fungus *Irpex lacteus* was cultivated on PDB medium for 7 days and was then subjected to a usual workup for extraction and isolation to yield four phenolic compounds (**5-27**–**5-30**) [30]. Two phenolic secondary metabolites (**5-28** and **5-31**) were isolated from the *Streptomyces coelescens* YT-2 from *G. elata* [54]. The strain *Alternaria* sp. H-35, an endophytic fungus of *G. elata*, was subjected to large-scale fermentation using medium, and a systematic study of the chemical constituents of its secondary metabolites was conducted, leading to the isolation of two phenolic compounds (**5-32** and **5-33**). In addition, compound **5-33** showed no antibacterial activity against *Staphylococcus aureus*, *Escherichia coli*, and *Candida albicans* [55]. A systematic chemical investigation of the secondary metabolites from the co-culture of *Phoma* sp. YUD17001 and *Armillari* led to the isolation of eight phenolic compounds (**5-2**, **5-28**, **5-33,** and **5-34**–**5-38**). From the liquid co-culture metabolites of the endophytic fungus *Epicoccum* sp. YUD17002 and *Armillaria mellea*, eight phenolic compounds (**5-39**–**5-46**) were isolated. From the endophytic fungus *Penicillium* sp. TG-46 from *G. elata*, four phenolic compounds (**5-32**, **5-47**–**5-49**) were isolated [41]. Duan et al. isolated and identified one phenolic compound (**5-38**) from the endophytic fungus *Penicillium* sp. T2-8. Moreover, using the endophytic fungus *Penicillium* sp. T2-8 from *G. elata* as the wild strain, effective mutant strains were screened and cultured through combined mutagenesis with ultraviolet (UV, λ = 360 nm) irradiation and the chemical mutagen nitrosoguanidine (NTG, 1-methyl-3-nitro-1-nitrosoguanidine). Among them, the mutant strain *Penicillium* sp. T2-M20 yielded six phenolic compounds (**5-50**–**5-55**) upon isolation. (E)-7-(2-hydroxy-4-(hydroxymethyl) phenyl)-2-methyloct-6-enoic acid (**5-56**) and 1-(2,6-dihydroxyphenyl) pentan-1-one (**5-57**) were isolated from the endophytic fungus *Penicillium* sp. T2-11 from *G. elata*. Four phenolic compounds (**5-5**, **5-10**, **5-58** and **5-59**) were isolated from the endophytic fungus *Aspergillus* sp. T2-19. In addition, one phenolic compound (**5-60**) was isolated from the endophytic fungus *Alternaria* TY2-3 associated with *G. elata*. which had no significant inhibitory activity against four pathogenic strains: *Escherichia coli*, *Staphylococcus aureus*, *Bacillus subtilis*, and *Candida albicans* [36].

### 5.6. Steroids

**Figure 9:**
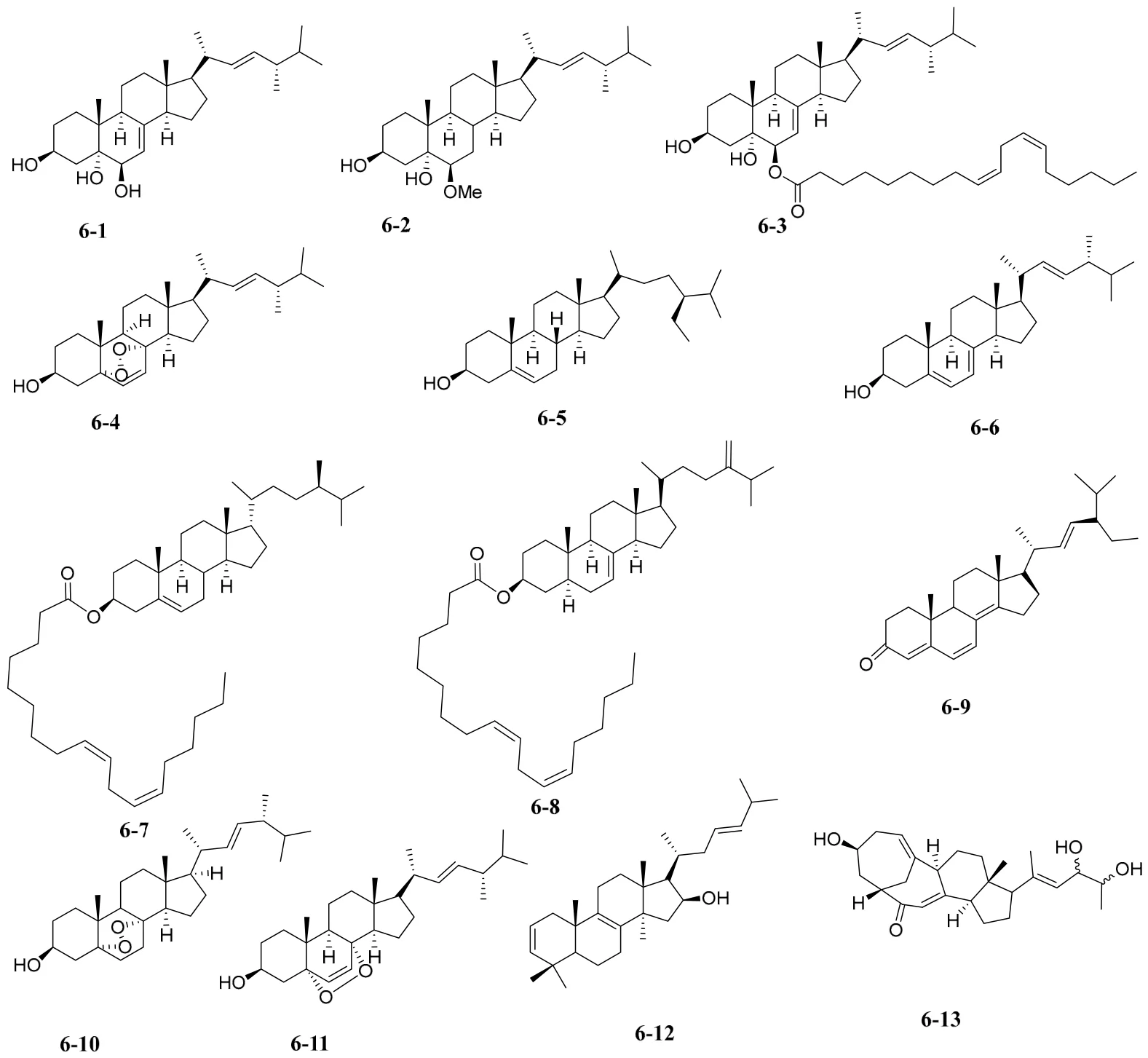
Steroids **6-1**–**6-13** in endophytic fungi of *G. elata.*

Steroids are a class of compounds containing the basic backbone structure of cyclopentane polyhydrophenanthrene with two horn methyl groups and C-17 side chains [101]. Steroids are produced by various organisms, including plants, animals, bacteria, and fungi, and they are responsible as protective agents for biotic and abiotic stress [102,103]. As shown in Figure 9 and Table 8, thirteen kinds of steroids and their derivatives (**6-1**–**6-13**) have been summarized. Among them, an endophytic fungus—*Schizophyllum commune*—was isolated from *G. elata* seeds in Zhaotong, Yunnan, from which three steroidal compounds (**6-1**–**6-3**) were isolated [29]. Two steroidal compounds (**6-1** and **6-4**) were isolated from the PDB liquid fermentation of *Aspergillus* sp. T2-19 and *Armillaria* sp., one of which was identical to compound **6-1**. One steroidal compound (**6-5**) was obtained from the rice solid-state fermentation of *Aspergillus* sp. T2-19 and *Armillaria* sp. [51]. One of the endophytic fungi isolated from *G. elata* collected in Zhaotong, Yunnan, identified as *Aspergillus cristatus*, was fermented, from which three steroidal compounds (**6-6**–**6-8**) were isolated [48]. Three steroidal compounds (**6-9**, **6-10** and **6-1**) were isolated from the endophytic fungus *Irpex lacteus* of *G. elata* cultured on PDA medium. The chemical investigation of the secondary metabolites from *Alternaria* sp. H-35 by large-scale fermentation of the rain using PDB medium was carried out, which led to the isolation of two steroidal compounds (**6-11** and **6-6**) [55]. One steroidal compound (**6-11**) was isolated from the co-culture broth of *Epicoccum* sp. YUD12 and *Armillaria* sp. [41]. Two steroidal compounds (**6-11** and **6-12**) were identified from the wild strain T2-8 after artificial mutagenesis to cillium. The investigation of the metabolites from endophyte *Penicilliu* sp. T2-11 cultured in host “Tianma” (*G. elata*) revealed two steroidal compounds (**6-11** and **6-13**) [36].

### 5.7. Fatty Acids

**Figure 10:**
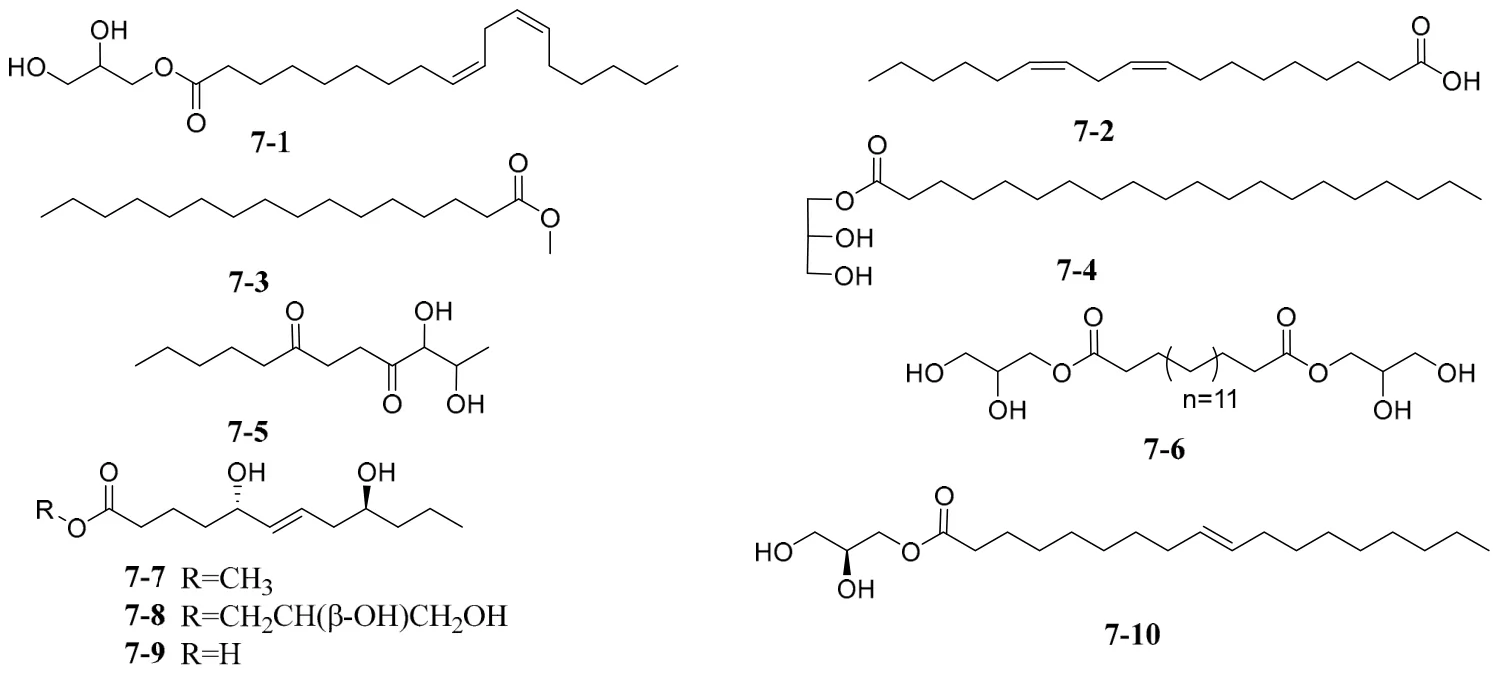
Fatty acid compounds **7-1**–**7-10** in endophytic fungi of *G. elata*.

Fatty acid compounds are important secondary metabolites of endophytic fungi of *G. elata*. The fatty-acid-type secondary metabolites and their biological activities of *G. elata* endophytes are shown in Table 9 and Figure 10. A fatty acid compound (**7-1**) was isolated and identified from *Schizophyllum commune*, an endophytic fungus of *G. elata* in Zhaotong, which has antifeedant activity against silkworms, with an antifeedant inhibition index (FDI) of 65%. In the antimicrobial activity assay, compound **7-1** showed inhibitory activity against *Beauveria bassiana*, MIC < 1 μg/mL [29]. An endophytic strain was isolated from *G. elata* and identified as *Aspergillus cristatus*. Three fatty acid compounds (**7-2**–**7-4**) were isolated and identified from its secondary metabolites, and none of the three compounds had antibacterial activity against *Escherichia coli* and *Staphylococcus aureus* [48]. A fatty acid compound (**7-5**) was isolated and identified from the endophytic fungus *Irpex lacteus* in *G. elata* seeds, and showed antifungal activity against *P. polonicum*, *T. atroviride*, and *P. subsingeri*, with MICs ≤ 32 μg/mL, but it did not show obvious antifungal activity against *Armillaria mellea* with the MICs 128 μg/mL [30]. Hylodiglyceride (**7-6**) was isolated and identified from the endophytic fungus *Alternaria* sp. H-35 [55]. Phytochemical investigation of the secondary metabolites of the co-culture system of endophytic fungi *Phoma* sp. YUD17001 and *Armillaria* sp. led to the isolation of two new fatty acid compounds, namely Phomesters A and B (**7-7** and **7-8**), together with one known analog (**7-9**). 9-octadecenoic acid-2′,3′- dihydroxypropyl ester (**7-10**) was isolated from the secondary metabolites of the co-culture system of endophyte *Epicoccum* sp. YUD17002 and *Armillaria* sp. [41].

### 5.8. Other

**Figure 11:**
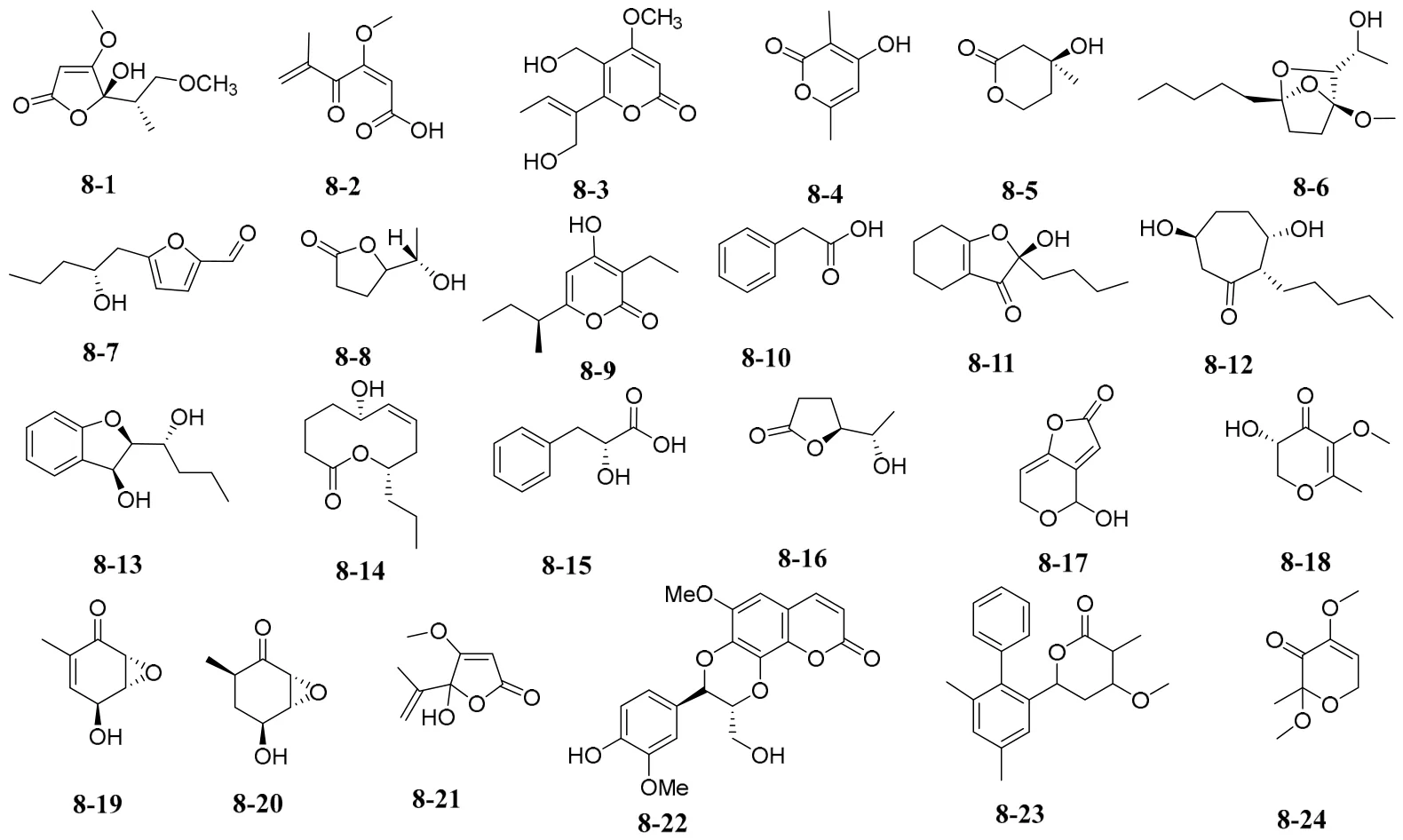
Other compounds **8-1**–**8-24** in endophytic fungi of *G. elata*.

At present, 24 other compounds (**8-1**–**8-24**) from the secondary metabolites of endophytic fungus have been isolated and identified, which are summarized in Table 10 and Figure 11. An in-depth study was conducted on the secondary metabolites of the gastrodia endophytic fungus *Penicillium skriabinii*, and a lactone compound (**8-1**) and a carboxylic acid compound (**8-2**) were isolated from it. Moreover, compound **8-2** has an inhibitory effect on acetylcholinesterase [35]. After solid culture of Gastrodia endophytic fungus *Arcopilus* sp. YUD20001, three lactone compounds (**8-3**–**8-5**) were isolated and identified from the secondary metabolites [53]. One ether compound (**8-6**), one furan derivative (**8-7**), and one lacidate compound (**8-8**) were isolated and identified from the secondary metabolites of the Gastrodia endophytic fungus *Irpex lacteus*. Compound **8-7** exhibited significant antimicrobial activity against *Penicillium polonicum*, with MICs of 8 μg/mL [30]. From the endophytic actinomycete *Streptomyces* genus YT-2, a lactone (**8-9**) and a benzene ring derivative (**8-10**) were isolated and identified [54]. Six compounds (**8-11**–**8-16**) were isolated and identified from the secondary metabolites co-cultured with *Gastrodia* endophytic fungus *Phoma* sp. YUD17001 and *Armillaria* sp. Among them, phomretone G (**8-11**) has moderate inhibitory activity against *E. coli* with an MIC of 8 µg/mL [41]. Four compounds (**8-17**–**8-20**) were isolated and identified from the metabolites of the gastrodia endophyte *Penicillium* sp. TG-46 [41]. A lactone compound (**8-21**) was isolated from the *Gastrodia* endophyte *Penicillium* sp. T2-8. Screening of mutant strains obtained after artificial mutagenesis of the wild strain *P.* sp. T2-8 revealed the metabolites of the mutant strain *Penicillium* sp. T2-M20, from which three compounds (**8-21**–**8-23**) were isolated and identified. One compound (**8-24**) was isolated and identified from the gastrodia endophytic fungus *Penicillium* sp. T2-11 [36].

## 6. Conclusions and Perspectives

Our investigation shows that endophytic community in *Gastrodia elata* is highly diverse and influenced by the season, location, and host tissue site. These fungal endophytes may be prospective producers of an abundant and dependable source of bioactive and chemically novel compounds. Notably, clear correlations exist between fungal taxonomic affiliation and metabolite skeleton types. Ascomycetes including *Penicillium*, *Aspergillus* and *Alternaria* mainly produce polyketides, anthraquinones and phenolic derivatives. By contrast, Basidiomycetes such as *Schizophyllum commune* and *Irpex lacteus* prefer to synthesize terpenoids and fatty acids with insect-resistant activity. In general, strains belonging to the same genus usually generate structurally similar compounds, while phylogenetically distant fungi yield highly differentiated metabolites.

Beyond conventional single strain cultivation, co-cultivation (especially co-culture with *Armillaria mellea*) has been proven to be the most effective strategy to induce novel secondary metabolites [104,105]. In a co-culture system, two strains grow together either symbiotically or in competition for nutrients and space, establishing an equilibrium population and lifestyle state [106]. Some examples of molecules derived from fungal co-cultures have been reported, including Cytochathiazines A–C [107], Amycolapeptins A and B [108], Chermebilaenes A and B [109]. Notably, most terpenoids with strong antifeedant activity and acetylcholinesterase inhibitory activity were obtained from co-culture systems in this review.

Nevertheless, existing research on *G. elata* endophytic fungi still presents several critical limitations that restrict in-depth resource utilization and pharmaceutical translation. First, merely a minor portion of endophytic isolates retrieved from the investigated habitat have undergone traditional isolation strategies, whereas countless unclassified and phylogenetically uncharacterized endophytic lineages persist underexplored, rendering the reservoir of endophytic microbes largely untapped. The current culture collection suffers from inadequate taxonomic breadth, with surveys interrogating endophytic species diversity, populational distribution dynamics, and the recovery of dominant strains lack systematic comprehensiveness. Secondly, metabolite extraction and profiling were only performed on a handful of identified isolates, leaving the majority of strains’ biosynthetic potential untapped. Even for tested strains, merely a few known metabolites were detected, without systematic dissection of metabolic profiles, biosynthetic pathways and chemical diversity. A vast array of bioactive specialized metabolites with promising functions remain undiscovered, and the metabolic capacity of endophytes is far from fully exploited. Thirdly, only a tiny fraction of strains and novel compounds underwent preliminary bioassays, while comprehensive activity validation and mechanistic exploration are absent. Most strains were not screened for antimicrobial, antioxidant, antitumor and growth-promoting capacities. Moreover, the potency, molecular targets, action mechanisms and applicable scenarios of identified metabolites have not been thoroughly characterized, making it impossible to clarify their practical value and bridge fundamental research to applied bioprospecting.

To address these bottlenecks, future research priorities should focus on the following directions. First, integrate culture-independent sequencing and metagenomic strategies to comprehensively explore unculturable fungal resources and complete the overall diversity profile of *G. elata* endophytes [110,111,112]. Second, apply genome-guided mining, biosynthetic gene cluster mining, metabolomics, and co-culture induction strategies to unlock silent biosynthetic pathways and excavate novel bioactive metabolites [113,114,115,116]. Third, strengthen in vivo pharmacological verification and molecular mechanistic studies to accurately evaluate the medicinal value of fungal compounds and avoid overestimation based merely on in vitro data. Finally, explore the ecological interactions between endophytic fungi and *G. elata* hosts to reveal the regulatory roles of fungal metabolites in plant physiological metabolism.

Collectively, *G. elata* endophytic fungi possess great resource potential, but their current research depth and industrial transformation remain limited. This review clarifies existing research deficiencies and provides targeted future prospects, aiming to promote further systematic exploration and sustainable utilization of *G. elata* endophytic fungal resources.

## Figures and Tables

**Table 2:** Biological activities of endophytes isolated from different parts of *G. elata.*

Endophytic	Culture Conditions	Extract	Activities	Ref.
*Herbaspirillum*	PDB, 28 °C, 24 h, shaking (120 r/min)	PDB medium	Antimicrobial	[37]
*Serratia* *plymuthica*	PDB, 28 °C, 24 h, shaking (120 r/min)	PDB medium	Antimicrobial	[37]
*Acremonium*	PDB, 28 °C, 24 h, shaking (120 r/min)	PDB medium	Antimicrobial	[37]
*Bacillus velezensis* (B1)	DMEM, (30 ± 1) °C, 3 d, no shaking	DMEM medium	Antimicrobial Antioxidant	[39]
*Bacillus velezensis* (B2)	DMEM, (30 ± 1) °C, 3 d, no shaking	DMEM medium	Antimicrobial Antioxidant Anticancer	[39]
*Lelliottia* sp.	DMEM, (30 ± 1) °C, 3 d, no shaking	DMEM medium	Antimicrobial Antioxidant Anticancer	[39]
*Rahnella* *aquatilis*	DMEM, (30 ± 1) °C, 3 d, no shaking	DMEM medium	Antimicrobial Antioxidant Anticancer	[39]
*Oidiodendron* sp.	PDA, 28 °C, 6–7 d, no shaking	Ethanol extraction	Antioxidant	[52]
*Neonectria* sp.	PDA, 28 °C, 6–7 d, no shaking	Ethanol extraction	Antioxidant	[52]
GER2	PDA, 28 °C, 6–7 d, no shaking	Ethanol extraction	Antioxidant	[52]
*Epicoccum* sp.	PDB, 28 °C, 7 d, shaking (150–200 rpm)	EtOAc extracts	Antifungal	[62]
*Pycnoporus* sp.	-, 2–7 d, shaking (100 rpm)	Fermentation liquid	Antioxidant	[58]

**Table 3:** Terpenoids **1-1**–**1-102** in endophytic fungi of *G. elata* and their biological activities.

Compounds	Strain	Biological Activities	Novelty	Structural Classification	Host Tissue	Ref.
Schimunin A (**1-1**)	*Schizophyllum commune*	Antifeedant activity	New	Bisabolane sesquiterpenes	Seed	[29]
Schimunin B (**1-2**)	*Schizophyllum commune*	Antifeedant activity	New	Bisabolane sesquiterpenes	Seed	[29]
Schimunin C (**1-3**)	*Schizophyllum commune*	Antifeedant activity	New	Bisabolane sesquiterpenes	Seed	[29]
Schimunin D (**1-4**)	*Schizophyllum commune*	Antifeedant activity	New	Bisabolane sesquiterpenes	Seed	[29]
Schimunin E (**1-5**)	*Schizophyllum commune*	Antifeedant activity	New	Bisabolane sesquiterpenes	Seed	[29]
Aleuriol (**1-6**)	*Schizophyllum commune*	Antifeedant activity	Known	Bisabolane sesquiterpenes	Seed	[29]
4-Dehydrodihy-dromelIeolide (**1-7**)	Co-cultivation of *Aspergillus* sp. T2-19 with *Armillaria*	-	Known	Sesquiterpenes	-	[51]
Armillarivin (**1-8**)	Co-cultivation of *Aspergillus* sp. T2-19 with *Armillaria*	-	Known	Sesquiterpenes	-	[51]
(*R*)-(-)-hydroxysydo nic acid (**1-9**)	Co-cultivation of *Aspergillus* sp. T2-19 with *Armillaria*	-	Known	Bisabolane sesquiterpenes	-	[51]
(*S*)-(-)-sydowic acid (**1-10**)	Co-cultivation of *Aspergillus* sp. T2-19 with *Armillaria*	-	Known	Bisabolane sesquiterpenes	-	[51]
Preaustinoid A1 (**1-11**)	*Penicillium skriabinii*	-	Known	Meroterpenes	Tuber	[35]
Preaustinoid A2 (**1-12**)	*Penicillium skriabinii*	-	Known	Meroterpenes	Tuber	[35]
1,2-dihydroacetoxy dehydroaustin (**1-13**)	*Penicillium skriabinii*	-	Known	Meroterpenes	Tuber	[35]
Dehydroausin (**1-14**)	*Penicillium skriabinii*	Acetylcholinesterase inhibitory activity	Known	Meroterpenes	Tuber	[35]
Austin (**1-15**)	*Penicillium skriabinii*	-	Known	Meroterpenes	Tuber	[35]
Irpexlactin A (**1-16**)	*Irpex lacteus*	Antifungal activity	New	Sesquiterpene glycoside	Seed	[30]
Irpexlactin B (**1-17**)	*Irpex lacteus*	Antifungal activity	New	Sesquiterpene	Seed	[30]
Irpexlactin C (**1-18**)	*Irpex lacteus*	-	New	Sesquiterpene	Seed	[30]
Irpexlactin D (**1-19**)	*Irpex lacteus*	-	New	Sesquiterpene	Seed	[30]
Irpexlactin E (**1-20**)	*Irpex lacteus*	-	New	Sesquiterpene	Seed	[30]
Irlactin G (**1-21**)	*Irpex lacteus*	Antifungal activity	Known	Sesquiterpene	Seed	[30]
Conocenol C (**1-22**)	*Irpex lacteus*	Antifungal activity	Known	Sesquiterpene	Seed	[30]
(3*S*,6*R*,7*R*)-tremul-1-ene-6,11,12-triol (**1-23**)	*Irpex lacteus*	-	Known	Sesquiterpene	Seed	[30]
11,12-dihydroxy-1-tremulen-5-one (**1-24**)	*Irpex lacteus*	-	Known	Sesquiterpene	Seed	[30]
Neroplofurol (**1-25**)	*Irpex lacteus*	Antifungal activity	Known	Sesquiterpene	Seed	[30]
11,12-epoxy-5,6-secotremula-1,6(13)- dien-5,12-olide (**1-26**)	*Irpex lacteus*	-	Known	Sesquiterpene	Seed	[30]
Tremulenediols A (**1-27**)	*Irpex lacteus*	-	Known	Sesquiterpene	Seed	[30]
11,12-epoxy-10α-hydroxy-5,6-seco-1,6(13)-tremuladien-5,12-olide (**1-28**)	*Irpex lacteus*	-	Known	Sesquiterpene	Seed	[30]
Ceriponol L (**1-29**)	*Irpex lacteus*	-	Known	Sesquiterpene	Seed	[30]
11,12-epoxy-12β-hydroxy-1-tremulen -5-one (**1-30**)	*Irpex lacteus*	-	Known	Sesquiterpene	Seed	[30]
Tricycloalternarene 3b (**1-31**)	*Alternaria* sp. H-35	Antimicrobial activity	Known	Meroterpenoid	-	[55]
Tricycloalternarene 2b (**1-32**)	*Alternaria* sp. H-35	-	Known	Meroterpenoid	-	[55]
Actg-toxine (**1-33**)	*Alternaria* sp. H-35	-	Known	Meroterpenoid	-	[55]
Armilaroma (**1-34**)	Co-culture of *Phoma* sp. YUD17001-*Armillaria* sp.	Cytotoxic, Acetylcholinesterase inhibitory, and Antibacterial activity	New	Protoilludane sesquiterpeniod	Tuber	[41]
4-dehydrodihy dromelleolide (**1-35**)	Co-culture of *Phoma* sp. YUD17001-*Armillaria* sp.	-	Known	Protoilludane sesquiterpeniod	Tuber	[41]
Armullane (**1-36**)	Co-culture of *Phoma* sp. YUD17001-Armillaria sp	-	Known	Protoilludane sesquiterpeniod	Tuber	[41]
10α-hydroxymell eolide (**1-37**)	Co-culture of *Phoma* sp. YUD17001- *Armillaria* sp.	-	Known	Protoilludane sesquiterpeniod	Tuber	[41]
Melleolide (**1-38**)	Co-culture of *Phoma* sp. YUD17001- *Armillaria* sp.	-	Known	Protoilludane sesquiterpeniod	Tuber	[41]
Armillaridin (**1-39**)	Co-culture of *Phoma* sp. YUD17001- *Armillaria* sp.	-	Known	Protoilludane sesquiterpeniod	Tuber	[41]
Ehydroarmillylorsellinate (**1-40**)	Co-culture of *Phoma* sp. YUD17001- *Armillaria* sp.	-	Known	Protoilludane sesquiterpeniod	Tuber	[41]
10-ahydroxymelle olide (**1-41**)	Co-culture of *Phoma* sp. YUD17001- *Armillaria* sp.	-	Known	Protoilludane sesquiterpeniod	Tuber	[41]
Armillol (**1-42**)	Co-culture of Phoma sp. YUD17001- *Armillaria* sp.	-	Known	Protoilludane sesquiterpeniod	Tuber	[41]
Epiterenoid A (**1-43**)	Co-culture of *Epicoccum* sp. YUD17002-*Armillaria* sp.	-	New	Polyoxygenated meroterpenoid	Tuber	[41]
Epiterenoid B (**1-44**)	Co-culture of *Epicoccum* sp. YUD17002-*Armillaria* sp.	-	New	Polyoxygenated meroterpenoid	Tuber	[41]
Epiterenoid C (**1-45**)	Co-culture of *Epicoccum* sp. YUD17002-*Armillaria* sp.	-	New	Polyoxygenated meroterpenoid	Tuber	[41]
Armiloid A (**1-46**)	Co-culture of *Epicoccum* sp. YUD17002-*Armillaria* sp.	Acetylcholinesterase inhibitory and Antimicrobial activity	New	Illudalane sesquiterpeniod	Tuber	[41]
Epicoterpene A (**1-47**)	Co-culture of *Epicoccum* sp. YUD17002-*Armillaria* sp.	Antimicrobial activity	New	Protoilludane sesquiterpeniod	Tuber	[41]
Epicoterpene B (**1-48**)	Co-culture of *Epicoccum* sp. YUD17002-*Armillaria* sp.	-	New	Protoilludane sesquiterpeniod	Tuber	[41]
Epicoterpene C (**1-49**)	Co-culture of *Epicoccum* sp. YUD17002-*Armillaria* sp.	Antimicrobial activity	New	Protoilludane sesquiterpeniod	Tuber	[41]
Epicoterpene D (**1-50**)	Co-culture of *Epicoccum* sp. YUD17002-*Armillaria* sp.	Antimicrobial activity	New	Protoilludane sesquiterpeniod	Tuber	[41]
Epicoterpene E (**1-51**)	Co-culture of *Epicoccum* sp. YUD17002-*Armillaria* sp.	-	New	Protoilludane sesquiterpeniod	Tuber	[41]
Armilliphatic A (**1-52**)	Co-culture of *Epicoccum* sp. YUD17002-*Armillaria* sp.	Cytotoxic, antimicrobial, and Acetylcholinesterase inhibitory activity	New	Protoilludane sesquiterpeniod	Tuber	[41]
Armilliphatic D (**1-53**)	Co-culture of Epicoccum sp. YUD17002-*Armillaria* sp.	-	New	Protoilludane sesquiterpeniod	Tuber	[41]
Armilliphatic E (**1-54**)	Co-culture of *Epicoccum* sp. YUD17002-*Armillaria* sp.	-	New	Protoilludane sesquiterpeniod	Tuber	[41]
Armillarivin (**1-55**)	Co-culture of *Epicoccum* sp. YUD17002-*Armillaria* sp.	-	Known	Protoilludane sesquiterpeniod	Tuber	[41]
Armillarin (**1-56**)	Co-culture of *Epicoccum* sp. YUD17002-*Armillaria* sp.	-	Known	Protoilludane sesquiterpeniod	Tuber	[41]
Melleolide K (**1-57**)	Co-culture of *Epicoccum* sp. YUD17002-*Armillaria* sp.	-	Known	Protoilludane sesquiterpeniod	Tuber	[41]
Melleolide L (**1-58**)	Co-culture of *Epicoccum* sp. YUD17002-*Armillaria* sp.	-	Known	Protoilludane sesquiterpeniod	Tuber	[41]
Armillaritin (**1-59**)	Co-culture of *Epicoccum* sp. YUD17002-*Armillaria* sp.	-	Known	Protoilludane sesquiterpeniod	Tuber	[41]
Melledonal (**1-60**)	Co-culture of *Epicoccum* sp. YUD17002-*Armillaria* sp.	-	Known	Protoilludane sesquiterpeniod	Tuber	[41]
5′-O-methylmelledonal (**1-61**)	Co-culture of *Epicoccum* sp. YUD17002-*Armillaria* sp.	-	Known	Protoilludane sesquiterpeniod	Tuber	[41]
10-dehydroxy-melleoliede B (**1-62**)	Co-culture of *Epicoccum* sp. YUD17002-*Armillaria* sp.	-	Known	Protoilludane sesquiterpeniod	Tuber	[41]
14-hydroxydihydromelleolide (**1-63**)	Co-culture of *Epicoccum* sp. YUD17002-*Armillaria* sp.	-	Known	Protoilludane sesquiterpeniod	Tuber	[41]
Echinocidin B (**1-64**)	Co-culture of *Epicoccum* sp. YUD17002-*Armillaria* sp.	-	Known	Protoilludane sesquiterpeniod	Tuber	[41]
Δ^6^-protoilluden (**1-65**)	Co-culture of *Epicoccum* sp. YUD17002-*Armillaria* sp.	-	Known	Protoilludane sesquiterpeniod	Tuber	[41]
Russujaponol H (**1-66**)	Co-culture of *Epicoccum* sp. YUD17002-*Armillaria* sp.	-	Known	Protoilludane sesquiterpeniod	Tuber	[41]
Guaidiol (**1-67**)	Co-culture of *Epicoccum* sp. YUD17002-*Armillaria* sp.	-	Known	Sesquiterpeniod	Tuber	[41]
Graphostromane E (**1-68**)	Co-culture of *Epicoccum* sp. YUD17002-*Armillaria* sp.	-	Known	Sesquiterpeniod	Tuber	[41]
Lutein (**1-69**)	Co-culture of *Epicoccum* sp. YUD17002-*Armillaria* sp.	-	Known	Tetraterpenoid	Tuber	[41]
Turbinaric acid (**1-70**)	Co-culture of *Epicoccum* sp. YUD17002-*Armillaria* sp.	-	Known	Tetraterpenoid	Tuber	[41]
(+)-trans-sobrerol (**1-71**)	Co-culture of *Epicoccum* sp. YUD17002-*Armillaria* sp.	-	known	Monoterpenoids	Tuber	[41]
(5β,6α)-6,11-dihydroxyeudesmane (**1-72**)	*Penicillium* sp. TG-46	-	Known	Sesquiterpenoid	-	[41]
Preaustinoid A6 (**1-73**)	*Penicillium* sp. T2-8	-	New	Meroterpenoid	Tuber	[36]
Dihydroxyneogrifolic acid (**1-74**)	*Penicillium* sp. T2-8	-	New	Neogrifolin derivative	Tuber	[36]
Preaustinoid A1 (**1-75**)	*Penicillium* sp. T2-8	Antibacterial activity	Known	Meroterpenoid	Tuber	[36]
Neoaustin (**1-76**)	*Penicillium* sp. T2-8	-	Known	Meroterpenoid	Tuber	[36]
Austin (**1-77**)	*Penicillium* sp. T2-8	-	Known	Meroterpenoid	Tuber	[36]
Preaustinoid A2 (**1-78**)	*Penicillium* sp. T2-8	-	Known	Meroterpenoid	Tuber	[36]
Neogrifolin (**1-79**)	*Penicillium* sp. T2-8	Cytotoxic and antibacterial activity	Known	Neogrifolin derivative	Tuber	[36]
Grifolin (**1-80**)	*Penicillium* sp. T2-8	-	Known	Neogrifolin derivative	Tuber	[36]
(*S*)-9-hydroxy-10,22-ene-neogrifolin (**1-81**)	*Penicillium* sp. T2-8	-	Known	Neogrifolin derivative	Tuber	[36]
(*S*)-18,19-dihydroxyneogrifolin (**1-82**)	Penicillium sp. T2-8	-	Known	Neogrifolin derivative	Tuber	[36]
Confluentin (**1-83**)	*Penicillium* sp. T2-8	-	Known	Neogrifolin derivative	Tuber	[36]
Peniterester (**1-84**)	*Penicillium* sp. T2-M20	Antibacterial activity	New	Sesquiterpenoid	-	[36]
Aspterric acid (**1-85**)	*Penicillium* sp. T2-M20	-	Known	Sesquiterpenoid	-	[36]
2(*S*)-hydroxyalbicanol-11-acetate (**1-86**)	*Penicillium* sp. T2-11	-	Known	Sesquiterpenoid	Tuber	[36]
Gaomastenol D (**1-87**)	*Penicillium* sp. T2-11	-	Known	Sesquiterpenoid	Tuber	[36]
Nardosinanols C (**1-88**)	*Penicillium* sp. T2-11	-	Known	Sesquiterpenoid	Tuber	[36]
Decumbenone C (**1-89**)	*Aspergillus* sp. T2-19	-	Known	Sesquiterpenoid	Tuber	[36]
Decumbenones A (**1-90**)	*Aspergillus* sp. T2-19	-	Known	Sesquiterpenoid	Tuber	[36]
Peaurantiogriseol F (**1-91**)	*Aspergillus* sp. T2-19	-	Known	Sesquiterpenoid	Tuber	[36]
Versiol (**1-92**)	*Aspergillus* sp. T2-19	-	Known	Sesquiterpenoid	Tuber	[36]
Actg-toxin D (**1-93**)	*Alternaria* sp. TY2-3	-	Known	Meroterpenoid	-	[36]
Actg-toxin E (**1-94**)	*Alternaria* sp. TY2-3	-	Known	Meroterpenoid	-	[36]
Tricycloalternarene D (**1-95**)	*Alternaria* sp. TY2-3	-	Known	Meroterpenoid	-	[36]
Tricycloalternarene 6b (**1-96**)	*Alternaria* sp. TY2-3	-	Known	Meroterpenoid	-	[36]
Tricycloalternarene 6A (**1-97**)	*Alternaria* sp. *TY2-3*	-	Known	Meroterpenoid	-	[36]
Tricycloalternarene 1A (**1-98**)	*Alternaria* sp. TY2-3	-	Known	Meroterpenoid	-	[36]
Tricycloalternarene B (**1-99**)	*Alternaria* sp. TY2-3	-	Known	Meroterpenoid	-	[36]
Guignarenone B (**1-100**)	*Alternaria* sp. TY2-3	-	Known	Meroterpenoid	-	[36]
Tricycloalterfurene A (**1-101**)	*Alternaria* sp. TY2-3	-	Known	Meroterpenoid	-	[36]
Monocycloalternarene A (**1-102**)	*Alternaria* sp. TY2-3	-	Known	Neogrifolin derivative	-	[36]

**Table 4:** Polyketides **2-1**–**2**-**37** in endophytic fungi of *G. elata* and their biological activities.

Compounds	Strain	Biological Activities	Novelty	Host Tissue	Ref.
Schimutone (**2-1**)	*Schizophyllum commune*	Antifeedant activity	New	Seed	[29]
Versiol (**2-2**)	Co-culture of *Aspergillus* sp. T2-19 and *Armillaria mellea*	-	Known	-	[51]
Isoversiol A (**2-3**)	Co-culture of *Aspergillus* sp. T2-19 and *Armillaria mellea*	-	Known	-	[51]
Alternariol (**2-4**)	*Arcopilus* sp. YUD20001	-	Known	-	[53]
Alternariol-9-o-monomethyl ether (**2-5**)	*Arcopilus* sp. YUD20001	-	Known	-	[53]
Alternariol-9-methyl ether (**2-6**)	*Alternaria* sp. H-35	-	Known	-	[55]
Alternariol (**2-7**)	*Alternaria* sp. H-35	-	Known	-	[55]
Phomretonea A (**2-8**)	Co-culture of *Phoma* sp. YUD17001 and *Armillaria* sp.	-	New	Tuber	[41]
Phomretonea B (**2-9**)	Co-culture of *Phoma* sp. YUD17001 and *Armillaria* sp.	-	New	Tuber	[41]
Phomretonea C (**2-10**)	Co-culture of *Phoma* sp. YUD17001 and *Armillaria* sp.	-	New	Tuber	[41]
Phomretonea D (**2-11**)	Co-culture of *Phoma* sp. YUD17001 and *Armillaria* sp.	-	New	Tuber	[41]
Phomretonea E (**2-12**)	Co-culture of *Phoma* sp. YUD17001 and *Armillaria* sp.	-	New	Tuber	[41]
Phomretonea F (**2-13**)	Co-culture of *Phoma* sp. YUD17001 and *Armillaria* sp.	-	New	Tuber	[41]
De-o-methyldiaporthin (**2-14**)	Co-culture of *Phoma* sp. YUD17001 and *Armillaria* sp.	-	Known	Tuber	[41]
(–)-5-methylmellein (**2-15**)	Co-culture of *Phoma* sp. YUD17001 and *Armillaria* sp.	-	Known	Tuber	[41]
Altechromone A (**2-16**)	Co-culture of *Phoma* sp. YUD17001 and *Armillaria* sp.	-	Known	Tuber	[41]
6-hydroxymellein (**2-17**)	*Penicillium* sp. T2-8	-	Known	Tuber	[36]
Djalonensone (**2-18**)	*Penicillium* sp. T2-M20	-	Known	-	[36]
Penicitritone A (**2-19**)	*Penicillium* sp. T2-11	Antimicrobial and cytotoxic activity	New	Tuber	[36]
Penicitritone B (**2-20**)	*Penicillium* sp. T2-11	Antimicrobial activity	New	Tuber	[36]
Asperbiphenyl (**2-21**)	*Penicillium* sp. T2-11	-	Known	Tuber	[36]
Citrinin (**2-22**)	*Penicillium* sp. T2-11	-	Known	Tuber	[36]
(3R*,4S*)-6,8-dihydroxy-3,4,7- Trimethylisocoumarin (**2-23**)	*Penicillium* sp. T2-11	-	Known	Tuber	[36]
2,3-dihydro-5-hydroxy-2-methylchromn-4-one (**2-24**)	*Penicillium* sp. T2-11	-	Known	Tuber	[36]
Citreoisocoumarin (**2-25**)	*Penicillium* sp. T2-11	-	Known	Tuber	[36]
3-methoxy-6,8-dihydroxy-3-methyl-3,4-dihydroisocoumarin (**2-26**)	*Alternaria* sp. TY2-3	-	Known	-	[36]
Talarophilone (**2-27**)	*Talaromyces* sp. YUD18002	-	New	Tuber	[47]
Epi-pinophilin B (**2-28**)	*Talaromyces* sp. YUD18002	-	Known	Tuber	[47]
Pinophilin B (**2-29**)	*Talaromyces* sp. YUD18002	-	Known	Tuber	[47]
Multioketide A (**2-30**)	*Penicillium* sp. YUD17006	-	New	-	[56]
Multioketide B (**2-31**)	*Penicillium* sp. YUD17006	-	New	-	[56]
Multioketide C (**2-32**)	*Penicillium* sp. YUD17006	Acetylcholinesterase and PTP1B inhibitory activity	New	-	[56]
O-menthylcurvulinic acid (**2-33**)	*Penicillium* sp. YUD17006	-	Known	-	[56]
Methyl 2-acetyl-3,5- Dihydroxyphenylacetate (**2-34**)	*Penicillium* sp. YUD17006	-	Known	-	[56]
Integrastatin B (**2-35**)	*Penicillium* sp. YUD17006	-	Known	-	[56]
Trichodenoid A (**2-36**)	*T* *. reesei*	-	New	-	[57]
Trichodenoid B (**2-37**)	*T* *. reesei*	Antioxidant	New	-	[57]

* Denotes relative configuration.

**Table 5:** Alkaloids **3-1**–**3-24** in endophytic fungi of *G. elata* and their biological activities.

Compounds	Strain	Biological Activities	Novelty	Host Tissue	Ref.
Brevianamide K (**3-1**)	Co-cultured of *Aspergillus* sp. T2-19 with *Armillaria* sp.	Antimicrobial and cytotoxic activity	Known	-	[51]
Brevianamide Q (**3-2**)	Co-cultured of *Aspergillus* sp. T2-19 with *Armillaria* sp.	-	Known	-	[51]
Brevianamide R (**3-3**)	Co-cultured of *Aspergillus* sp. T2-19 with *Armillaria* sp.	Cytotoxic activity	Known	-	[51]
Verruculogen (6) (**3-4**)	*Penicillium skriabinii*	Antifeedant and antimicrobial activity	Known	Tuber	[35]
(5aS,12S,14aS) -5a, 6-dihydroxy-12 -(2-hydroxy-2-methylpropyl)-9- methoxy-1,2,3,5a,6,14a–hexahydropyrrolo [1″,2″:4′,5′] pyrazino [1′,2′:1,6] pyrido [3, 4-b]indole-5, 14 (11H,12H)-dione (**3-5**)	*P*. *skriabinii*	-	Known	Tuber	[35]
Cyclotryprostatin A (8) (**3-6**)	*P* *. skriabinii*	Antimicrobial activity	Known	Tuber	[35]
Cyclo-L-tryptophyl-L- proline (**3-7**)	*P* *. skriabinii*	*-*	Known	Tuber	[35]
Arcopiniol C (**3-8**)	*Arcopilus* sp. YUD20001	-	New	-	[53,92]
2-phenylacetamide (**3-9**)	*Arcopilus* sp. YUD20001	-	Known	-	[53]
N-hydroxy-2-(2-hydroxyphenyl) acetamide (**3-10**)	*Arcopilus* sp. YUD20001	-	Known	-	[53]
Indole-3-carboxaldehyde (**3-11**)	*Arcopilus* sp. YUD20001	-	Known	-	[53]
(+)-Didymellamide B (**3-12**)	*Arcopilus* sp. YUD20001	-	Known	-	[53]
1H-indazole (**3-13**)	*Arcopilus* sp. YUD20001	-	Known	-	[53]
Cyclo-(L-Pro-L-Ile) (**3-14**)	*Streptomyces coelescens*	-	Known	-	[54]
Cyclo-(L-Pro-L-Phe) (**3-15**)	*Streptomyces coelescens*	-	Known	-	[54]
Thymidine (**3-16**)	*Alternaria* sp. H-35	-	Known	-	[55]
Phomester C (**3-17**)	Co-cultivation of *Phoma* sp. YUD17001 with *Armillaria mellea*	-	New	Tuber	[41,93]
4-aminophenol (**3-18**)	Co-cultivation of *Phoma* sp. YUD17001 with *Armillaria mellea*	-	Known	Tuber	[41]
Uridine (**3-19**)	Co-cultivation of *Epicoccum* sp. YUD17002 with *Armillaria mellea*	-	Known	Tuber	[41]
Nosine (**3-20**)	*Penicillium* sp. T2-M20	-	Known	-	[36]
GKK1032 (**3-21**)	*Penicillium* sp. T2-11	-	Known	Tuber	[36]
Indol-3-carbaldehyde (**3-22**)	*Penicillium* sp. T2-11	-	Known	Tuber	[36]
Epi-deoxybrevianamide E (**3-23**)	*Aspergillus* sp. T2-19	-	Known	-	[36]
3-carbaldehydes (**3-24**)	*Aspergillus* sp. T2-19	-	Known	-	[36]

**Table 6:** Anthraquinones **4-1**–**4-26** in endophytic fungi of *G. elata* and their biological activities.

Compounds	Strain	Biological Activities	Novelty	Host Tissue	Ref.
Sterigmatocystin (**4-1**)	Co-culture of *Aspergillus* sp. T2-19 with *Armillaria* sp.	-	Known	-	[51]
Oxisterigmatocystin D (**4-2**)	Co-culture of *Aspergillus* sp. T2-19 with *Armillaria* sp.	-	Known	-	[51]
6,8-di-o-methylaverufin (**4-3**)	Co-culture of *Aspergillus* sp. T2-19 with *Armillaria* sp.	-	Known	-	[51]
Averufin (**4-4**)	Co-culture of *Aspergillus* sp. T2-19 with Armillaria sp.	Cytotoxic activity	Known	-	[51]
6-O-methylaverufin (**4-5**)	Co-culture of *Aspergillus* sp. T2-19 with *Armillaria* sp.	-	Known	-	[51]
6,8-di-o-methylnidurufin (**4-6**)	Co-culture of *Aspergillus* sp. T2-19 with *Armillaria* sp.	Cytotoxic and antimicrobial activity	Known	-	[51]
Versicolorin B (**4-7**)	Co-culture of *Aspergillus* sp. T2-19 with *Armillaria* sp.	Cytotoxic and antimicrobial activity	Known	-	[51]
8-O-methylversicolorin (**4-8**)	Co-culture of *Aspergillus* sp. T2-19 with *Armillaria* sp.	-	Known	-	[51]
Averisin (**4-9**)	Co-culture of A*spergillus* sp. T2-19 with *Armillaria* sp.	Cytotoxic and antimicrobial activity	Known	-	[51]
Versicolorin A (**4-10**)	Co-culture of *Aspergillus* sp. T2-19 with *Armillaria* sp.	-	Known	-	[51]
Averythrin (**4-11**)	Co-culture of *Aspergillus* sp. T2-19 with *Armillaria* sp.	-	Known	-	[51]
6,8,1′-tri-O-methylaverantin (**4-12**)	Co-culture of *Aspergillus* sp. T2-19 with *Armillaria* sp.	Cytotoxic activity	Known	-	[51]
Parietinic acid (**4-13**)	Co-culture of *Aspergillus* sp. T2-19 with *Armillaria* sp.	-	Known	-	[51]
Arugosin C (**4-14**)	Co-culture of *Aspergillus* sp. T2-19 with *Armillaria* sp.	Cytotoxic activity	Known	-	[51]
Questin (**4-15**)	*Aspergillus cristatus* T2 -10	-	Known	-	[48]
Altersolanol L (**4-16**)	*Arcopilus* sp. YUD20001	-	Known	-	[53]
(4S)-3,4-Dihydro-4,8-dihydroxy-l(2H)-naphthaleno (**4-17**)	Co-culture of *Phoma* sp. YUD17001 with *Armillaria* sp.	-	Known	Tuber	[41]
2-methoxy-6-ethyl-1,4-benzoquinone (**4-18**)	*Penicillium* sp. T2-8	-	Known	Tuber	[36]
2-methoxy-6-methylcyclohexa-2,5-diene-1,4-dione (**4-19**)	*Penicillium* sp. T2-8	-	Known	Tuber	[36]
2-methoxy-6-benzyl-1,4-benzoquinone (**4-20**)	*Penicillium* sp. T2-8	-	Known	Tuber	[36]
1-o-(2,4-dihydroxy-6-methylbenzoyl)-glycerol (**4-21**)	*Penicillium* sp. T2-M20	-	Known	-	[36]
Huperxanthones B (**4-22**)	*Penicillium* sp. T2-11	-	Known	Tuber	[36]
Sterigmatocystin (**4-23**)	*Aspergillus* sp. T2-19	-	Known	-	[36]
O-methylsterigmatocystin (**4-24**)	*Aspergillus* sp. T2-19	-	Known	-	[36]
Averufin (**4-25**)	*Aspergillus* sp. T2-19	-	Known	-	[36]
6,8,1′-tri-O-methylaverantin (**4-26**)	*Aspergillus* sp. T2-19	-	Known	-	[36]

**Table 7:** Phenolics **5-1**–**5-60** in endophytic fungi of *G. elata* and their biological activities.

Compounds	Strain	Biological Activities	Novelty	Host Tissue	Ref.
1-(4-hydroxyphenyl)propan-2-one (**5-1**)	*Schizophyllum commune*	Antifeedant activity	Known	Seed	[29]
Orsellinic acid (**5-2**)	Co-cultivation of *Aspergillus* sp. T2-19 with *A. mellea*	-	Known	-	[51]
Orcinol (**5-3**)	Co-cultivation of *Aspergillus* sp. T2-19 with *A. mellea*	-	Known	-	[51]
2-hydroxy phenyl acetic acid (**5-4**)	Co-cultivation of *Aspergillus* sp. T2-19 with *A. mellea*	-	Known	-	[51]
Diorcinol (**5-5**)	Co-cultivation of *Aspergillus* sp. T2-19 with *A. mellea*	-	Known	-	[51]
4-carboxydiorcinal (**5-6**)	Co-cultivation of *Aspergillus* sp. T2-19 with *A.* mellea	-	Known	-	[51]
Acetylfunicin (**5-7**)	Co-cultivation of *Aspergillus* sp. T2-19 with *A. mellea*	-	Known	-	[51]
Violaceol I (**5-8**)	Co-cultivation of *Aspergillus* sp. T2-19 with *A. mellea*	-	Known	-	[51]
Violaceol II (**5-9**)	Co-cultivation of *Aspergillus* sp. T2-19 with *A. mellea*	-	Known	-	[51]
Sydowiol D (**5-10**)	Co-cultivation of *Aspergillus* sp. T2-19 with *A. mellea*	-	Known	-	[51]
Sydowiol B (**5-11**)	Co-cultivation of *Aspergillus* sp. T2-19 with *A. mellea*	-	Known	-	[51]
Diorcinol D (**5-12**)	Co-cultivation of *Aspergillus* sp. T2-19 with *A. mellea*	-	Known	-	[51]
Diorcinol E (**5-13**)	Co-cultivation of *Aspergillus* sp. T2-19 with *A. mellea*	-	Known	-	[51]
Diorcinol K (**5-14**)	Co-cultivation of *Aspergillus* sp. T2-19 with *A. mellea*	-	Known	-	[51]
(α,S)-4-Hydroxy-2-(3-hydroxy-5- methylphenoxy)-6-methyl-α-(1-Meth- ylethenyl) benzeneethanol (**5-15**)	Co-cultivation of *Aspergillus* sp. T2-19 with *A. mellea*	-	Known	-	[51]
Diorcinol B (**5-16**)	Co-cultivation of *Aspergillus* sp. T2-19 with *A. mellea*	-	Known	-	[51]
(*S*)-5-(3-hydroxy-5-methylphenoxy)-2,2,7-trimethylchroman-3-ol (**5-17**)	Co-cultivation of *Aspergillus* sp. T2-19 with *A. mellea*	-	Known	-	[51]
Arcopiniols A (**5-18**)	*Arcopilus* sp. YUD20001	-	New	-	[53]
Arcopiniols B (**5-19**)	*Arcopilus* sp. YUD20001	Acetylcholinesterase inhibitory activity	New	-	[53]
Zinniol (**5-20**)	*Arcopilus* sp. YUD20001	-	Known	-	[53]
8-zinniol methyl ether (**5-21**)	*Arcopilus* sp. YUD20001	-	Known	-	[53]
Maristachone B (**5-22**)	*Arcopilus* sp. YUD20001	-	Known	-	[53]
(2S,3S)-3-hydroxybutan-2-yl 2-(4-hydroxyphenyl) acetate (**5-23**)	*Arcopilus* sp. YUD20001	-	Known	-	[53]
Methyl 4-hydroxyphenyl acetate (**5-24**)	*Arcopilus* sp. YUD20001	-	Known	-	[53]
1-(3-methoxy-4-methylphenyl) ethanone (**5-25**)	*Arcopilus* sp. YUD20001	-	Known	-	[53]
4-hydroxyphenylacetic acid (**5-26**)	*Arcopilus* sp. YUD20001	-	Known	-	[53]
2,5-dihydroxybenzoic acid (**5-27**)	*Irpex lacteus*	-	Known	Seed	[30]
P-hydroxy benzaldehyde (**5-28**)	*I. lacteus*	-	Known	Seed	[30]
Threo-4-hydroxyphenylpropan-7,8-diol (**5-29**)	*I. lacteus*	-	Known	Seed	[30]
Erythro-4-hydroxyphenylpropan-7,8-diol (**5-30**)	*I. lacteus*	-	Known	Seed	[30]
Methyl 4-hydroxyphenylacetate (**5-31**)	*Streptomyces coelescens* YT-2	-	Known	-	[54]
M-cresol (**5-32**)	*Alternaria* sp. H-35	-	Known	-	[55]
α-Acetylorcinol (**5-33**)	*Alternaria* sp. H-35	-	Known	-	[55]
Phexandiol A (**5-34**)	Co-cultivation of *Phoma* sp. YUD17001 with *Armillaria* sp.	-	New	Tuber	[41]
Scytalone (**5-35**)	Co-cultivation of *Phoma* sp. YUD17001 with *Armillaria* sp.	-	Known	Tuber	[41]
(3S)-3,6,7-trihydroxy-α-tetralon (**5-36**)	Co-cultivation of *Phoma* sp. YUD17001 with *Armillaria* sp.	-	Known	Tuber	[41]
46-dimethylcurvulinic acid (**5-37**)	Co-cultivation of *Phoma* sp. YUD17001 with *Armillaria* sp.	-	Known	Tuber	[41]
2-(4-hydroxyphenyl)-ethanol (**5-38**)	Co-cultivation of *Phoma* sp. YUD17001 with *Armillaria* sp.	-	Known	Tuber	[41]
Armilliphatic B (**5-39**)	Co-cultivation of *Epicoccum* sp. YUD17002 with *Armillaria* sp.	-	New	Tuber	[41]
Armilliphatic C (**5-40**)	Co-cultivation of YUD17002 with *Armillaria* sp.	-	New	Tuber	[41]
5-o-methylorsellinic acid (**5-41**)	Co-cultivation of YUD17002 with *Armillaria* sp.	-	Known	Tuber	[41]
6-chloroeverninic acid (**5-42**)	Co-cultivation of YUD17002 with *Armillaria* sp.	-	Known	Tuber	[41]
Isoevernin aldehyde (**5-43**)	Co-cultivation of YUD17002 with *Armillaria* sp.	-	Known	Tuber	[41]
2-hydroxy-4-methoxy-6-methylbenzaldehyde (**5-44**)	Co-cultivation of YUD17002 with *Armillaria* sp.	-	Known	Tuber	[41]
1,3-dihydro-7-methyl-4,6-isobenzofurandiol (**5-45**)	Co-cultivation of YUD17002 with *Armillaria* sp.	-	Known	Tuber	[41]
(+)-montagnetol (**5-46**)	Co-cultivation of YUD17002 with *Armillaria* sp.	-	Known	Tuber	[41]
3-methylbenzene-1,2-diol (**5-47**)	*Penicillium* sp. TG-46	-	Known	-	[41]
3-(hydroxymethyl) phenol (**5-48**)	*Penicillium* sp. TG-46	-	Known	-	[41]
Gentisyl alcohol (**5-49**)	*Penicillium* sp. TG-46	-	Known	-	[41]
Penicilone E (**5-50**)	*Penicillium* sp. T2-M20	-	Known	-	[36]
Aspersclerotiorone F(**5-51**)	*Penicillium* sp. T2-M20	-	Known	-	[36]
Aspergispiroketal (**5-52**)	*Penicillium* sp. T2-M20	-	Known	-	[36]
1-o-(2,4-dihydroxy-6-methylbenzoyl)-glycerol (**5-53**)	*Penicillium* sp. T2-M20	-	Known	-	[36]
16-β-hydroxylanosta-2,8,23-trien (**5-54**)	*Penicillium* sp. T2-M20	-	Known	-	[36]
Methyl-2,4-dihydroxy -3,5,6-trimethyl benzoate (**5-55**)	*Penicillium* sp. T2-M20	-	Known	-	[36]
(E)-7-(2-hydroxy-4-(hydroxymethyl) phenyl)- 2-methyloct-6-enoic acid (**5-56**)	*Penicillium* sp. T2-11	-	Known	Tuber	[36]
1-(2,6-dihydroxyphenyl) pentan-1-one (**5-57**)	*Penicillium* sp. T2-11	-	Known	Tuber	[36]
Cordyol C (**5-58**)	*Aspergillus* sp. T2-19	-	Known	-	[36]
(S)-(+)-sydonic acid (**5-59**)	*Aspergillus* sp. T2-19	-	Known	-	[36]
Altenusin (**5-60**)	*Alternaria* sp. TY2-3	-	Known	-	[36]

**Table 8:** Steroids **6-1**–**6-13** in endophytic fungi of *G. elata* and their biological activities.

Compounds	Strain	Biological Activities	Novelty	Host Tissue	Ref.
(3β, 5α, 6β)-ergosta-7,22- diene-3,5,6-triol (**6-1**)	*Schizophyllum commune*	-	Known	Seed	[29]
(3β, 5α, 6β, 22E)-6- methoxyergosta-7,22-diene-3,5-diol (**6-2**)	*S. commune*	-	Known	Seed	[29]
(3β, 5α, 6β)-ergosta-7,22- diene-3,5,6-triol-6-linoleate (**6-3**)	*S. commune*	-	Known	Seed	[29]
Ergosterol peroxide (**6-4**)	Co-cultivation of *Aspergillus* sp. T2-19 with *A. mellea*	-	Known	-	[51]
β-sitosterol (**6-5**)	Co-cultivation of *Aspergillus* sp. T2-19 with *A. mellea*	-	Known	-	[51]
Ergosterol (**6-6**)	*Aspergillus cristatus* T2-10	-	Known	-	[48]
β-sitosteryl linoleate (**6-7**)	*A. cristatus* T2-10	-	Known	-	[48]
3β-linoleyloxyergosta-7,24(28)-diene (**6-8**)	*A. cristatus* T2-10	-	Known	-	[48]
Choleta-4,6,8(14),22-tetraen-3-one (**6-9**)	*Irpex lacteus*	-	Known	Seed	[30]
Ergosterol peroxide (**6-10**)	*I. lacteus*	-	Known	Seed	[30]
Ergosterol endoperoxide (**6-11**)	*Alternaria* sp. H-35	-	Known	-	[55]
16β-Hydroxylanosta-2,8,23-trien (**6-12**)	*Penicillium* sp. T2-M20	-	Known	-	[36]
Neocyclocitrinols A (**6-13**)	*Penicillium* sp. T2-11	-	Known	Tuber	[36]

**Table 9:** Fatty acid compounds **7-1**–**7-10** in endophytic fungi of *G. elata* and their biological activities.

Compounds	Strain	Biological Activities	Novelty	Host Tissue	Ref.
1-O-(9Z,12Z-octadecadienoyl)-glycerol (**7-1**)	*Schizophyllum commune*	Antifeedant and antimicrobial activity	Known	Seed	[29]
Linoleic acid (**7-2**)	*Aspergllus cristatus*	-	Known	-	[48]
N-hexadecanoic acid methylester (**7-3**)	*A. cristatus*	-	Known	-	[48]
Eicosanoic acid monoglyceride (**7-4**)	*A. cristatus*	-	Known	-	[48]
2,3-dihydroxydodacane-4,7-dione (**7-5**)	*Irpex lacteus*	Antifungal activity	Known	Seed	[30]
Hylodiglyceride (**7-6**)	*Alternaria* sp. H-35	-	Known	-	[55]
Phomester A (**7-7**)	Co-culture *Phoma* sp. YUD17001 with *Armillaria* sp.	-	New	Tuber	[41]
Phomester B (**7-8**)	Co-culture *Phoma* sp. YUD17001 with *Armillaria* sp.	-	New	Tuber	[41]
(E)-5,9-dihydroxydodec-6-enoic acid (**7-9**)	Co-culture *Phoma* sp. YUD17001 with *Armillaria* sp.	-	Known	Tuber	[41]
9-octadecenoic acid-2′,3′- dihydroxypropyl ester (**7-10**)	Co-culture *Epicoccum* sp. YUD17002 with *Armillaria* sp.	-	Known	Tuber	[41]

**Table 10:** Other compounds **8-1**–**8-24** in endophytic fungi of *G. elata* and their biological activities.

Compounds	Strain	Biological Activities	Novelty	Host Tissue	Ref.
6-methoxy-5 (6) -dihydropenicillic acid (**8-1**)	*Penicillium skriabinii*	-	Known	Tuber	[35]
Penicillic acid (**8-2**)	*P.* skriabinii	-	Known	Tuber	[35]
Chaetoglocin A (**8-3**)	*Arcopilus* sp. YUD20001	Acetylcholinesterase inhibitory activity	Known	-	[53]
4-hydroxy-3,6-dimethyl-2-pyrone (**8-4**)	*Arcopilus* sp. YUD20001	-	Known	-	[53]
(R)-mevalonolactone (**8-5**)	*Arcopilus* sp. YUD20001	-	Known	-	[53]
Irpexlacin (**8-6**)	*Irpex lacteus*	-	New	Seed	[30]
Irpexlacte B (**8-7**)	*I. lacteus*	Antimicrobial activity	Known	Seed	[30]
(4*R*,5*S*)-5-hy-droxyhexan-4-olide (**8-8**)	*I. lacteus*	-	Known	Seed	[30]
Germicidin (**8-9**)	*Streptomyces coelescens* YT-2	-	Known	-	[54]
Phenylacetic acid (**8-10**)	*S. coelescens* YT-2	-	Known	-	[54]
Phomretonea G (**8-11**)	Co-culture of *Phoma* sp. YUD17001 with *Armillaria* sp.	Antimicrobial activity	New	Tuber	[41]
Phomretonea H (**8-12**)	Co-culture of *Phoma* sp. YUD17001 with *Armillaria* sp.	-	New	Tuber	[41]
Phexandiols B (**8-13**)	Co-culture of *Phoma* sp. YUD17001 with Armillaria sp.	-	New	Tuber	[41]
Putaminoxin (**8-14**)	Co-culture of *Phoma* sp. YUD17001 with *Armillaria* sp.	-	Known	Tuber	[41]
Phenyllactic acid (**8-15**)	Co-culture of *Phoma* sp. YUD17001 with *Armillaria* sp.	-	Known	Tuber	[41]
(4*S*,5*S*)-5-hydroxy-4-hexanolide (**8-16**)	Co-culture of *Phoma* sp. YUD17001 with *Armillaria* sp.	-	Known	Tuber	[41]
Patulin (**8-17**)	*Penicillium* sp. TG-46	-	Known	-	[41]
Streptopyrone (**8-18**)	*Penicillium* sp. TG-46	-	Known	-	[41]
(+)-epiepoformin (**8-19**)	*Penicillium* sp. TG-46	-	Known	-	[41]
(+)-dihydroepiepoformin (**8-20**)	*Penicillium* sp. TG-46	-	Known	-	[41]
Penicillic acid (**8-21**)	*Penicillium* sp. T2-8	-	Known	Tuber	[36]
Cleomiscosin A (**8-22**)	*Penicillium* sp. T2-M20	-	Known	-	[36]
Nigerapyrones B (**8-23**)	*P.* sp. T2-M20	-	Known	-	[36]
2,4-dimethoxy-2-methyl-6H-pyran-3-one (**8-24**)	*Penicillium* sp. T2-11	-	Known	Tuber	[36]

## Data Availability

All relevant data are included within the article.
